# Enhanced Ionic Conductivity at the Solid Electrolyte Interphase of Oxygen‐Doped Li_6_PS_5_Cl

**DOI:** 10.1002/advs.76563

**Published:** 2026-07-13

**Authors:** Sojeong Yang, Sungwoo Kang, Atefeh Yadegarifard, Hyun‐Jae Lee, Nongnuch Artrith, Jung‐Hoon Lee, Byungju Lee

**Affiliations:** ^1^ Computational Science Research Center Korea Institute of Science and Technology (KIST) Seoul Republic of Korea; ^2^ Nanoscience and Technology KIST School University of Science and Technology Seoul Republic of Korea; ^3^ Materials Chemistry and Catalysis Debye Institute for Nanomaterials Science Utrecht University Utrecht the Netherlands; ^4^ KU‐KIST Graduate School of Converging Science and Technology Korea University Seoul Republic of Korea

**Keywords:** argyrodite electrolytes, ionic conductivity, machine learning interatomic potential, oxygen doping, phase identification, phase stability, solid electrolyte interphase

## Abstract

Understanding the formation and transport properties of the solid electrolyte interphase (SEI) at the Li metal | solid state electrolyte (SSE) interface remains a central challenge for all‐solid‐state batteries, as this buried interphase governs interfacial resistance yet is largely inaccessible to direct experimental characterization. In this work, we combine machine‐learned interatomic potential molecular dynamics (MLIP–MD) simulations with a machine–learning phase identification framework to resolve kinetically formed SEI phases at the Li_6_PS_5‐x_ClO_x_ | Li interface and to elucidate the role of oxygen doping. Our simulations reveal that the dominant SEI formed at both the undoped and oxygen‐doped interfaces is a Li_2_S_1‐x‐y_P_0.5x_Cl_0.5x_O_y_ phase, corresponding to an anion‐substituted Li_2_S structure. Moderate oxygen incorporation into the SEI enhances ionic conductivity, whereas excessive oxygen doping degrades both bulk electrolyte conductivity and SEI thermodynamic stability. This trade‐off provides an atomistic explanation for the experimentally observed non‐monotonic dependence of interfacial performance on oxygen content.

## Graphical abstract

Machine‐learned molecular dynamics and machine‐learning‐based phase identification reveal the kinetically formed SEI at buried Li | Li_6_PS_5_Cl interfaces. The SEI is dominated by Li_2_S‐based anion‐substituted phases, while oxygen doping enhances SEI ionic conductivity. However, excessive oxygen destabilizes SEI phases and reduces bulk solid‐state electrolyte ionic conductivity, highlighting an optimal doping window for stable, conductive interfaces.

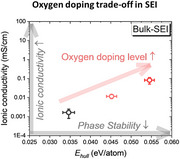

## 1 Introduction

The pursuit of high‐energy‐density and intrinsically safe electrochemical energy storage has driven intensive research into all‐solid‐state batteries. Replacing flammable liquid electrolytes with solid‐state electrolytes (SSEs) mitigates fire safety risks while enabling the integration of lithium metal anodes for high energy densities. Among various SSE candidates, sulfide‐based electrolytes, particularly the argyrodite‐type materials such as Li_6_PS_5_Cl, have attracted significant attention owing to their high room‐temperature ionic conductivity (∼1–10 mS/cm [1, 2]), ease of processing, and good mechanical flexibility. Moreover, the ionic conductivity of argyrodite electrolytes can be further enhanced through strategies such as introducing Li vacancies, altering the halogen distributions [3], or elemental doping [4, 5].

Despite their high ionic conductivity, argyrodite‐type sulfide electrolytes still face several critical challenges. First, they readily react with moisture in the air, releasing toxic H_2_S gas and decomposing. According to the hard and soft acid‐base theory [6, 7], the substitution of S^2−^ or P^5+^ by harder O^2−^ or suitable cation dopants can reduce the reactivity toward water and thereby improve the air stability of sulfide electrolytes, as demonstrated for various O‐containing [5, 8] or P‐free sulfide [9] systems. Second, interfacial instability remains a major challenge, particularly at the Li metal | argyrodite interface. At this boundary, a solid electrolyte interphase (SEI) layer forms, which can hinder Li ion transport, increase interfacial resistance, and potentially induce non‐uniform Li‐deposition and dendrite formation.

While the fundamental thermodynamic driving forces for interphase formation and the electrochemical stability window of the Li‐SSE interface are well described from density functional theory (DFT) [10, 11, 12], recent work has shown that metastable lithiated argyrodite phases can temporarily stabilize the interface and extend the apparent stability window beyond thermodynamic predictions [13]. The spatial distribution and transport properties of these metastable phases critically determine whether the SEI evolves into a passivating layer or a continuously growing, unstable one. Capturing the dynamic evolution of such SEIs under operating conditions requires simulations that can describe both atomistic reaction mechanisms and long‐time diffusion phenomena, which remain challenging for conventional ab initio molecular dynamics (AIMD) due to its high computational cost and limited accessible time and length scales.

Recent advances in machine‐learning interatomic potentials (MLIPs) [14] have enabled atomistic simulations of reactive Li | SSE interfaces [15, 16, 17, 18, 19, 20] and the formation of kinetically evolving SEI layers at scales inaccessible to conventional AIMD. For example, deep potential molecular dynamics (DeePMD) simulations of the Li | *β*‐Li_3_PS_4_ interface have elucidated a stepwise SEI formation mechanism, including ion interdiffusion, nucleation, Li_2_S growth, and stabilization [18]. For Li | Li_6_PS_5_Cl interface, DeePMD combined with enhanced sampling has revealed that metallic Li clusters nucleate within the Li_2_S/Li_3_P/LiCl‐rich SEI, and that the amorphous SEI exhibits a collapsed bandgap with finite electronic conductivity, thereby providing a pathway for electron leakage and Li reduction within the SEI [16]. Furthermore, for the Li | Li_7_P_3_S_11_ system, an atomic cluster expansion (ACE)‐based MLIP enables large‐scale simulations that capture the formation of a heterogeneous layered interphase containing nanocrystalline Li_2_S domains embedded in an amorphous matrix, and show that interphase growth gradually passivates as the ionic fluxes of Li, P, and S diminish [19]. These examples demonstrate that MLIP‐based MD can resolve the kinetics of SEI formation.

Building on these developments, we focus here on the role of oxygen doping in argyrodite‐type Li_6_PS_5‐x_ClO_x_ solid electrolytes. While O doping is known to enhance the air stability of sulfide electrolytes, its impact on interfacial reactions with Li metal and SEI formation has yet to be quantified. To capture not only thermodynamics but also the dynamics of SEI evolution, we construct an MLIP for the Li | Li_6_PS_5‐x_ClO_x_ interface and perform large‐scale MD simulations for both undoped and O‐doped systems. We then apply a machine‐learning‐based phase identification model to quantitatively classify SEI phases at the atomic level, thereby resolving how oxygen‐induced local environments correlate with interfacial transport properties and phase stability. This integrated approach – combining MLIP‐MD with automated phase identification – provides atomistic insight into how oxygen doping modulates the chemical and kinetic properties of the Li | Li_6_PS_5‐x_ClO_x_ interface. In particular, our simulations offer an atomistic explanation for why only moderate O doping leads to improved interfacial performance in experiments, thereby providing an atomistic basis for rational design guidelines for next‐generation sulfide‐based all‐solid‐state batteries.

## 2 Machine Learned Interatomic Potential for Modeling Li_6_PS_5‐x_ClO_x_ | Li Interfaces

To develop MLIPs for understanding the role of oxygen doping in different interface systems, we used the SevenNet model [21], which adopts the NequIP architecture [22], an E(3)‐equivariant GNN interatomic potential. Details of the training dataset and sampling strategy are provided in Section S1. The trained model achieved energy RMSE of 7.3 and 7.0 meV/atom for the training and validation sets, respectively, while the corresponding force RMSE values were 0.124 and 0.135 eV/Å.

**FIGURE 1:**
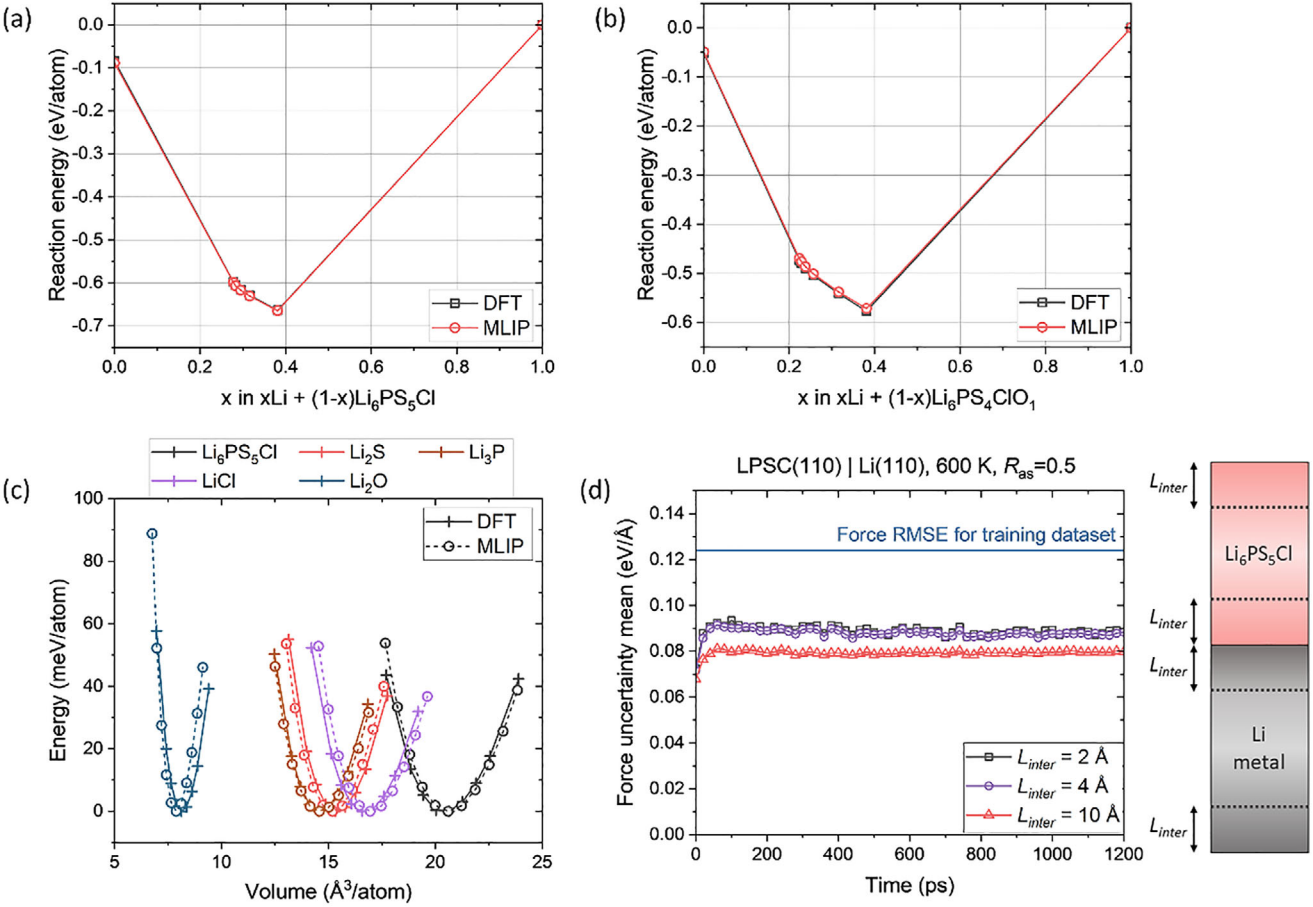
Reaction energies of (a) Li and Li_6_PS_5_Cl and (b) Li and Li_6_PS_4_ClO_1_ using DFT and MLIP. (c) Equations of state for possible decomposition products at the Li | Li_6_PS_5‐x_ClO_x_ interface (Li_6_PS_5_Cl, Li_2_S, Li_3_P, LiCl, and Li_2_O) calculated by DFT and MLIP. (d) Force uncertainty analysis in the interface region using an ensemble of five MLIPs trained with different random initializations. The time origin (0 ps) corresponds to the point immediately after the 100 ps equilibration run used to increase the temperature from 300 to 600 K.

We next validated the trained MLIP. Figure 1a,b shows a comparison of the reaction energies between Li and Li_6_PS_5_Cl, and between Li and Li_6_PS_4_ClO_1_, calculated by DFT and MLIP. The decomposition products and corresponding reaction energies are provided in Tables S3 and S4 in Section S3. The reaction energies, evaluated based on the thermodynamic stability of the decomposition products, are well reproduced by our MLIP. This indicates that our MLIP accurately describes the energy of possible decomposition products that may form during the interfacial reactions. Figure 1c compares the equations of state (EOS) calculated using DFT and MLIP for the thermodynamically predicted decomposition products (Li_6_PS_5_Cl, Li_2_S, Li_3_P, LiCl, and Li_2_O). The close agreement across a wide range of volumes demonstrates that our MLIP can reliably predict energies even far from the equilibrium volume, implying its robustness in capturing the large volume changes expected during interfacial reactions. Figure 1d shows the force uncertainty estimated using an ensemble approach, where five independently trained MLIPs with different initial parameters were used. The force uncertainty of atom *i* is defined as

(1) $$\sigma_{i}=\sqrt{\frac{1}{n}\sum_{k=1}^{n}{\left\|F_{i}^{k}-{\bar{F}}_{i}\right\|}^{2}}$$

where *n* is the number of MLIPs used in the ensemble (*n* = 5), $F_{i}^{k}$ is the force on atom *i* predicted by the *k* ^th^ MLIP, and ${\bar{F}}_{i}$ is the ensemble‐averaged force. Here, *F_i_* = (*F*_*i*,*x*_,  *F*_*i*,*y*_,  *F*_*i*,*z*_). To assess MLIP reliability near the interface, we computed the mean force uncertainty by averaging σ_*i*_ over atoms located within a defined interfacial region of thickness *L_inter_*. The resulting mean force uncertainty for atoms in the interfacial region remained below 0.1 eV/Å across all tested interface thicknesses (*L_inter_* = 2, 4, and 10 Å), confirming the reliability of our MLIP in describing interfacial reactions. For the interface thicknesses of *L_inter_* = 2, 4, 10 Å, the number of atoms included in the uncertainty averaging were 961, 1920, and 4773, respectively.

## 3 MLIP‐MD Simulations of SEI Formation at the Li_6_PS_5‐x_ClO_x_ | Li Interface

**FIGURE 2:**
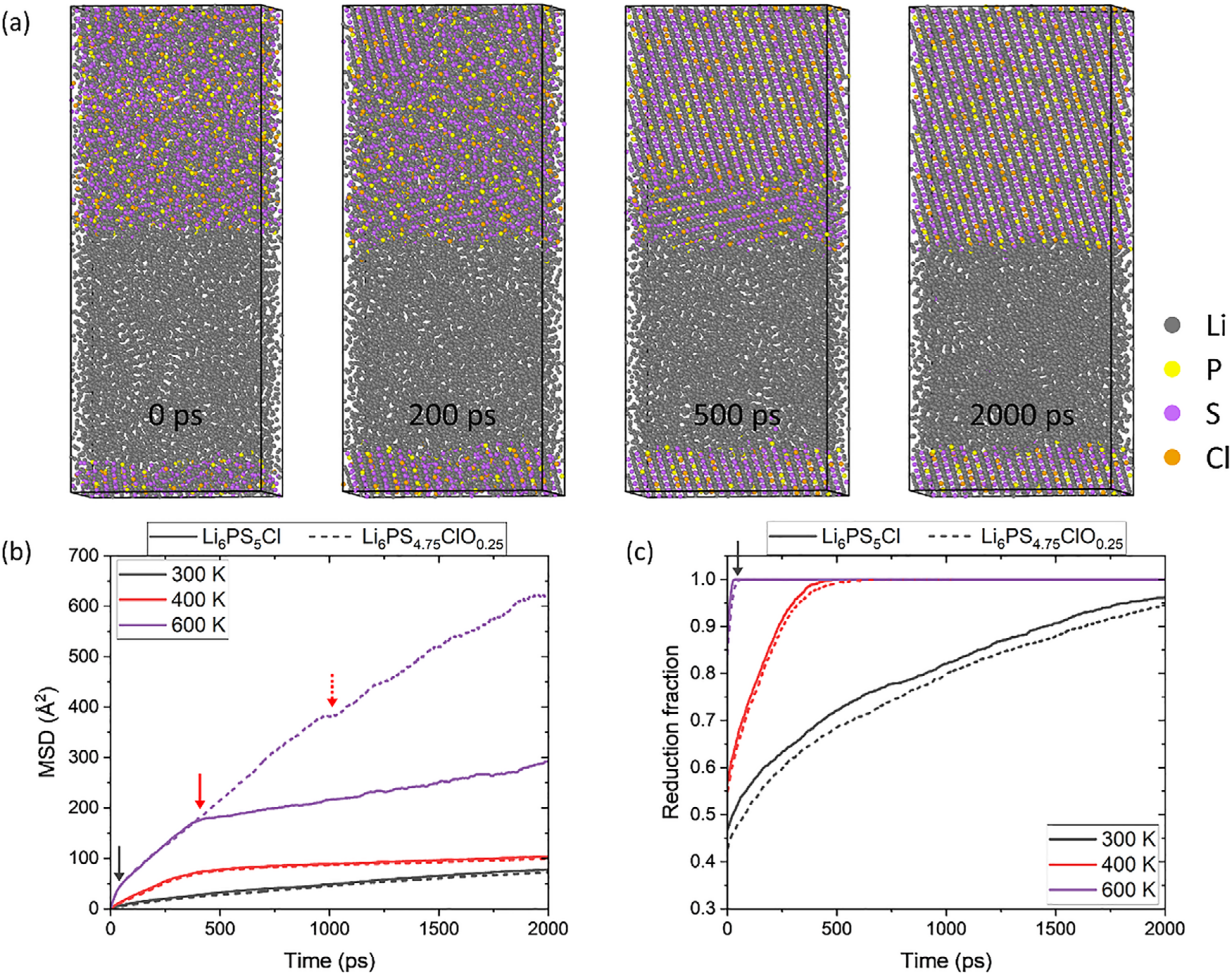
(a) Atomic structures of the Li_6_PS_5_Cl(110) | Li(110) interface at 600 K simulated under the NPT ensemble at 1000 bar. 0 ps indicates the initial structure of the production run, which is obtained after an equilibration run for heating from 300 K to the target temperature (300, 400, and 600 K). The structures were energy‐minimized at each timestep with fixed volume to clearly reveal the formed crystalline phases. (b) MSD of Li ions at the Li_6_PS_5‐x_ClO_x_ (110) | Li (110) interface (x = 0 and 0.25). The transition points from the fast to moderate diffusion regime and from the moderate to slow diffusion regime are indicated by black and red arrows, respectively. (c) Reduction fraction of the Li_6_PS_5‐x_ClO_x_ electrolyte during MD simulations at 300, 400, and 600 K.

Using the constructed MLIP, we performed MD simulations of the Li_6_PS_5‐x_ClO_x_(110) | Li(110) interface (x = 0.0, 0.25, and 0.5) to investigate interfacial reactions and SEI formation. Figure 2a visualizes the structural evolution of the Li_6_PS_5_Cl(110) | Li(110) interface at 600 K as a representative example after 100 ps of equilibration run (see Methods Section for details). Notably, a substantial fraction of the solid electrolyte had already decomposed during the equilibration run, resulting in an amorphous‐like interfacial structure at the beginning of the production run (*t* = 0).

Figure 2b shows the mean squared displacement (MSD) of Li ions originally in the SSE (excluding those in the Li metal). At 600 K, the MSD curve reveals three distinct diffusion regimes: (1) a fast diffusion regime, corresponding to the reduction‐driven breakdown of Li_6_PS_5‐x_ClO_x_; (2) a moderate diffusion regime, associated with structural reorganization and crystallization; and (3) a slow diffusion regime, characteristic of Li ion transport within the fully crystallized SEI.

The fast diffusion regime is closely linked to chemical reduction, which is driven by electron transfer from Li metal into Li_6_PS_5_Cl due to the higher Fermi level of Li metal. As shown in Figure 2c, the reduction fraction (*f_r_*) (defined in the Methods Section) increases monotonically; *f_r_* = 1 corresponds to a fully reduced state of Li_6_PS_5_Cl, i.e., when P^5+^ species in Li_6_PS_5_Cl are completely reduced to P^3 –^. Importantly, the onset of the moderate diffusion regime occurs at the same time that *f_r_* reaches unity: in other words, the fast to moderate diffusion transition coincides with the completion of reduction (*f_r_* = 1). This coincidence is observed at approximately 40 ps at 600 K and 400 ps at 400 K for both Li_6_PS_5_Cl and Li_6_PS_4.75_ClO_0.25_. At 300 K, full reduction was not achieved within the 2 ns simulation window (*f_r_* < 1), and therefore only the initial fast diffusion regime was observed.

The moderate diffusion regime corresponds to the crystallization of the SEI. In Figure 2a, the structures at 200 ps (within the moderate diffusion regime) already exhibit partial ordering. This regime terminates at around 400 ps, and the snapshot at 500 ps shows that the SEI has already reached a nearly fully crystallized structure. The resulting crystalline phase remains stable up to 2000 ps, and Li ion diffusion within this ordered SEI corresponds to the slow diffusion regime. At lower temperatures, crystallization is kinetically slowed; extended 8 ns simulations at 300 and 400 K (Section S5) confirm that the same diffusion regime behavior and dominant SEI phase are reproduced, but shifted to longer timescales. At 400 K, all three regimes appear, whereas at 300 K, the slow diffusion regime is not reached within the simulation window.

Regarding oxygen doping, Figure 2c shows that oxygen incorporation does not significantly change the reduction rate. However, it does affect crystallization kinetics. In the O‐doped interface (Li_6_PS_4.75_ClO_0.25_(110) | Li(110)), crystallization is delayed and largely completed only after around 1050 ps. This characteristic time is indicated by a dashed red arrow in Figure 2b, corresponding to the point at which the MSD slope changes from the moderate diffusion regime to the slow diffusion regime. This change in the MSD slope is more clearly observed for P, S, and Cl atoms (see Section S4). Consistently, the phase fraction analysis also shows nearly converged behavior beyond around 1050 ps, which will be discussed in Section 4. Interestingly, even after the majority of the SEI has crystallized (∼1050 ps), the Li MSD slope in the O‐doped SEI becomes larger than that of the undoped case, indicating higher Li‐ion diffusivity in the oxygen‐containing SEI. This trend is in contrast to the bulk behavior, where O doping slightly reduces Li‐ion diffusivity (Figure 4a), consistent with experimental observations [23]. To clarify why oxygen incorporation enhances Li ion diffusion in the SEI but not in the bulk, the subsequent section analyzes the phase composition and structural characteristics of the SEI using machine‐learning‐based phase identification.

## 4 Automated Phase Identification of SEI via Machine‐Learning Classification

**FIGURE 3:**
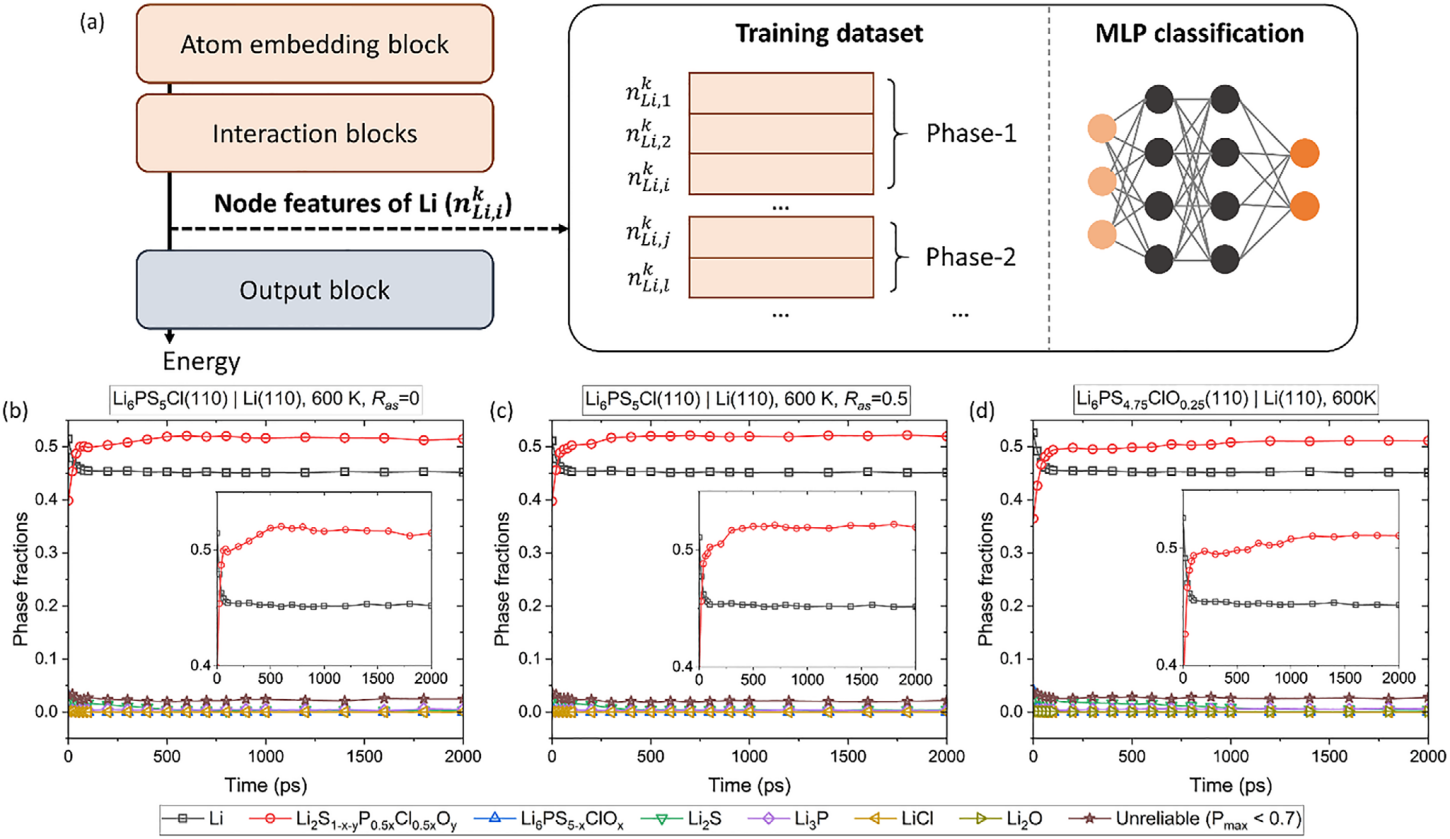
(a) Schematic illustration of the machine‐learning‐based phase identification workflow. A simplified NequIP architecture [22] used in SevenNet is shown, along with Li‐centered node features extracted from the final hidden layer, which are used as input features for the MLP classifier. The node features of Li atoms ($n_{Li,i}^{k}$), paired with their corresponding phase labels, constitute the training dataset. (b–d) Time evolution of the phase fractions predicted by the classifier during a 2 ns MD simulation performed under the NPT ensemble at 1000 bar and 600 K for (b) Li_6_PS_5_Cl(110) | Li(110) with *R_as_* = 0, (c) Li_6_PS_5_Cl(110) | Li(110) with *R_as_* = 0.5, and (d) O‐doped Li_6_PS_4.75_ClO_0.25_(110) | Li(110). Structures sampled during the MD simulations were energy‐minimized at fixed volume before using the ML‐based phase identification model. The insets in (b–d) show magnified views of the phase fraction of the Li_2_S_1‐x‐y_P_0.5x_Cl_0.5x_O_y_ phase.

To identify the SEI phases formed at the interface during the simulation, we constructed a machine‐learning based phase identification classifier. Different phases exhibit distinct Li‐centered local environments, characterized by the types of neighboring atoms as well as their interatomic distances and angles. To encode such complex local environments, we use the graph neural network (GNN) node embeddings of Li atoms, obtained from SevenNet, as feature vectors. In SevenNet, the node features in the final hidden layer represent refined embeddings obtained through multiple message‐passing iterations. During each message‐passing step, a node aggregates geometric and chemical information from its neighboring atoms and updates its feature vector. As a result, the final node embedding provides an expressive representation of the atom's local environment, which is subsequently used to predict atomic energies in the MLIP, as schematically illustrated in Figure 3a.

Accordingly, we used the Li‐centered node embeddings extracted from each phase as input features, assigned phase labels to the corresponding Li atoms, and trained a multilayer perceptron (MLP) classifier for phase identification (see Methods Section). Possible reaction products of Li_6_PS_5_Cl with Li, obtained from the Materials Project database [24], were included in the training set. In addition, we incorporated Li_2_S‐based substitutional phases, proposed in previous studies [17], in which P and Cl partially replace S sites in the Li_2_S structure (Li_2_S_1‐x_P_0.5x_Cl_0.5x_). The corresponding atomic structure is shown in Figure S21a. Although equilibrium thermodynamics predicts Li_2_S, LiCl, and Li_3_P as the final decomposition products, the formation of phase‐separated LiCl and Li_3_P requires long‐range migration and aggregation of Cl and P species. Such processes are expected to be kinetically limited. Consistent with this expectation, our MD simulations show that P and Cl remain nearly immobile even at 600 K (see Section S4), while Li_2_S‐type environments dominate at the interface (see Figure S10 in Section S7). Together with experimental reports that small amounts of foreign anions, such as Se and Te, can be doped into Li_2_S while preserving the Li_2_S‐type structure [25], these observations support the plausibility of Li_2_S‐based substitutional phases as SEI components formed during interfacial reactions. Further justification for including the Li_2_S_1‐x_P_0.5x_Cl_0.5x_ phase in the training set is provided in Section S7, where we analyze the phase fraction predictions obtained using an MLP classifier trained without this phase and present a t‐SNE [26] visualization of the embedding space to assess the representativeness of the training set. For the Li_6_PS_5_Cl and Li_2_S_1‐x_P_0.5x_Cl_0.5x_ phases, oxygen‐doped structures were also included in the training set. Undoped and O‐doped Li_6_PS_5_Cl structures were collectively labeled as Li_6_PS_5‐x_ClO_x_, and undoped and O‐doped Li_2_S_1‐x_P_0.5x_Cl_0.5x_ structures were grouped under the label Li_2_S_1‐x‐y_P_0.5x_Cl_0.5x_O_y_. A complete list of all training structures is provided in Section S6.

Figure 3b–d shows the time evolution of the phase fractions predicted by the ML‐based phase identification model during the 2 ns production run at 600 K for each interface system. Figure 3b,c correspond to the Li_6_PS_5_Cl(110) | Li(110) interface with anti‐site defect ratios *R_as_* = 0 and 0.5, respectively. The anti‐site defect originates from the exchange between S ions on the 4d sites and Cl ions on the 4a sites; *R_as_* = 1 represents the fully exchanged structure in which all S ions on 4d sites and all Cl ions on 4a sites are swapped. Motivated by previous studies that the bulk ionic conductivity of Li_6_PS_5_Cl can vary by up to two orders of magnitude at 300 K depending on the anti‐site defect ratio [27, 28], we compared *R_as_* = 0 and 0.5 to examine the influence of anti‐site disorder at the interface. In contrast to bulk behavior, the interfacial reaction shows almost no dependence on the anti‐site ratio. This is because Li_6_PS_5_Cl is highly unstable against reduction at the Li metal interface and rapidly decomposes, such that structural variations that would otherwise enhance Li‐ion transport in the bulk have negligible influence on the interface reaction kinetics. In other words, once the electrolyte is reduced, the anti‐site defect no longer determines the resulting SEI composition or its transport characteristics.

Figure 3d presents the phase fractions obtained for the O‐doped Li_6_PS_4.75_ClO_0.25_(110) | Li(110) interface. Similar to the undoped case, O‐doping does not lead to the formation of separate oxide phases such as Li_2_O; instead, O remains incorporated in substitutional Li_2_S‐type environments together with P and Cl. Consequently, the overall phase evolution is nearly identical to that observed for the undoped Li_6_PS_5_Cl(110) | Li(110) interface. For both undoped and O‐doped systems, the dominant SEI product is identified as Li_2_S_1‐x‐y_P_0.5x_Cl_0.5x_O_y_, which we hereafter refer to as the bulk‐SEI. Notably, the fraction of the bulk‐SEI phase converges between 1000 and 1200 ps, corresponding to the transition from the moderate diffusion regime to the slow diffusion regime discussed in Section 3. In addition, the Li_6_PS_5‐x_ClO_x_ SSE phase persists only for the first 40 ps before disappearing, which is consistent with the time when the reduction fraction becomes 1, as shown in Figure 2c.

## 5 Enhanced Ionic Conductivity of SEI Through Oxygen Doping

**FIGURE 4:**
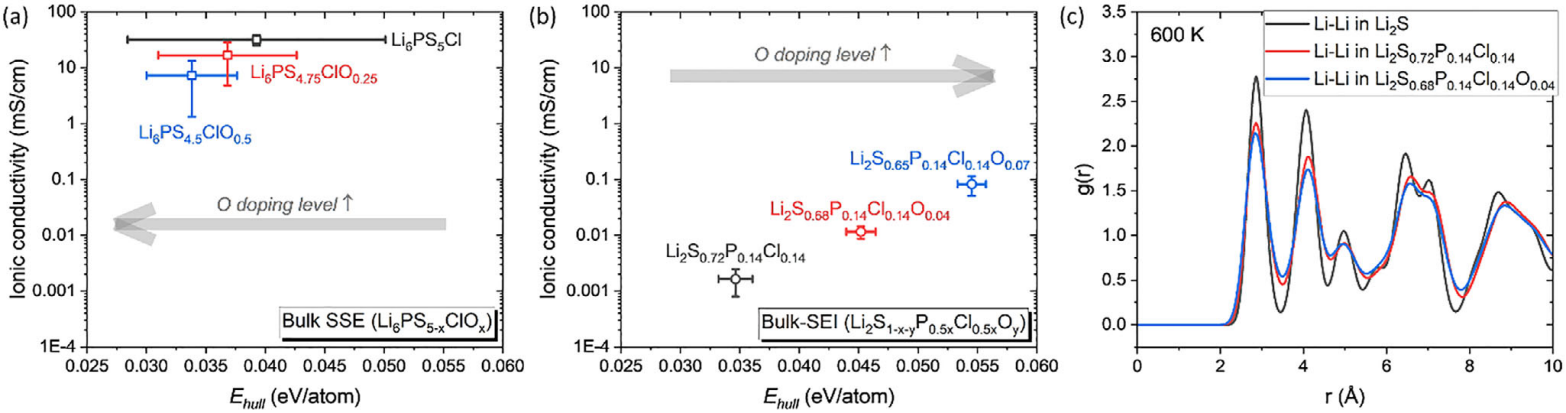
Calculated ionic conductivity at 300 K vs. energy above hull (*E_hull_*) for (a) O‐doped solid‐state electrolyte (SSE) Li_6_PS_5‐x_ClO_x_ and (b) the corresponding bulk‐SEI compositions Li_2_S_0.72‐x_P_0.14_Cl_0.14_O_x_. The bulk‐SEI compositions represent the reaction products of the SSE with Li metal, preserving the S:P:Cl:O ratio. Identical colors indicate corresponding O‐doping levels between the SSE and bulk‐SEI. Li‐ion diffusion coefficients were extrapolated to 300 K using Arrhenius fits to MD data at 600, 700, and 800 K, and the corresponding ionic conductivities were then calculated. For the bulk‐SEI, data points represent the mean values and standard deviations from five random configurations. (c) Radial distribution functions (RDFs) of Li‐Li pairs in Li_2_S, undoped bulk‐SEI (Li_2_S_0.72_P_0.14_Cl_0.14_), and O‐doped bulk‐SEI (Li_2_S_0.68_P_0.14_Cl_0.14_O_0.04_).

Having identified the dominant SEI composition formed at the Li metal interface, we next examine how O doping influences the ionic transport properties of both the parent SSE and the resulting bulk‐SEI phase. Figure 4a,b presents the calculated ionic conductivities at 300 K plotted against the energy above hull (*E_hull_*) for the bulk SSE (Li_6_PS_5‐x_ClO_x_) and the corresponding bulk‐SEI (Li_2_S_0.72‐x_P _0.14_Cl_0.14_O_x_), respectively. The *E_hull_* values were calculated using our MLIP trained on optB88‐vdw DFT data. In this calculation, solid oxides (e.g., Li_2_O) were considered as the relevant competing phases instead of gaseous O_2_, thereby avoiding the well‐known overbinding of O_2_ in GGA‐based functionals. As a result, the systematic oxygen‐related errors are largely cancelled when comparing relative thermodynamic stabilities among solid phases [29].

For the SSE (Figure 4a), multiple structures corresponding to different anti‐site defect ratios (*R_as_* = 0.0, 0.25, 0.5, 0.75, and 1.0) were considered for each O concentration. Here, *R_as_* denotes the anti‐site defect ratio associated with S(4d)‐Cl(4a) site exchange, a factor known to influence the ionic conductivity of the bulk SSE. The relative energies (*ΔE*) of these anti‐site defect structures were calculated (see Section S9), and Boltzmann weights at 300 K were applied to determine the weighted means and standard deviations for both ionic conductivity and *E_hull_*. This approach accounts for the thermal coexistence of multiple anti‐site defect configurations. As shown in Figure 4a, increasing O doping leads to a slight decrease in the ionic conductivity of the bulk SSE, while the *E_hull_* remains largely unchanged within the standard deviation. This trend in ionic conductivity aligns with the experimental observations of Indrawan et al. [23], who reported a reduction in bulk ionic conductivity with increasing O content in Li_6_PS_5‐x_ClO_x_.

In contrast, the bulk‐SEI phase exhibits markedly different behavior. For each O‐doping level, five independent bulk‐SEI structures with the composition Li_2_S_0.72‐x_P_0.14_Cl_0.14_O_x_ were generated by randomly assigning P, Cl, and O anions while preserving the overall S:P:Cl:O anion ratio derived from the parent SSE (see Section 9.3). The ionic conductivities and *E_hull_* were evaluated for each structure, with the resulting mean values and standard deviations shown in Figure 4b. Unlike the SSE, the bulk‐SEI phase shows a pronounced enhancement in ionic conductivity with increasing O content. Relative to the undoped composition (Li_2_S_0.72_P_0.14_Cl_0.14_), the ionic conductivity increases by approximately one order of magnitude at x = 0.04 in Li_2_S_0.72‐x_P _0.14_Cl_0.14_O_x_ and by nearly two orders of magnitude at x = 0.07, as illustrated in Figure 4b. Figure 4b also reveals a clear correlation between ionic conductivity and thermodynamic stability. While O doping significantly enhances Li‐ion transport, it concurrently increases *E_hull_*, indicating a reduction in the thermodynamic stability of the bulk‐SEI phase. This result highlights a trade‐off between enhanced ionic conductivity and phase stability in the SEI.

To gain insight into the origin of the enhanced Li‐ion diffusivity in Li_2_S_0.72‐x_P_0.14_Cl_0.14_O_x_ compared to pristine Li_2_S, radial distribution functions (RDFs) were analyzed. As shown in Figure 4c, the Li‐Li RDF peak becomes broader upon substitution of S with P, Cl, and O, indicating increased local structural disorder. The Li‐anion RDFs of Li_2_S_0.72_P_0.14_Cl_0.14_ and Li_2_S_0.68_P_0.14_Cl_0.14_O_0.04_, presented in Section S10, further reveal that substitutional doping shifts the Li‐Cl peaks to longer distances and the Li‐O peaks to shorter distances relative to Li‐S peak in Li_2_S. These changes reflect increased local lattice distortion. These results suggest that the introduction of foreign anions creates a more frustrated energy landscape for Li ions, enabling a greater diversity of migration pathways and facilitating enhanced Li‐ion diffusion. In particular, O doping, owing to its strong size mismatch relative to S, induces more pronounced local distortions, which are consistent with the additional increase in ionic conductivity observed. This claim is further supported by Li probability density maps and a site‐resolved hopping analysis (Section S10), which show that Li sites coordinated by O atoms undergo markedly more frequent site exchange than O‐free environments.

Overall, these results imply that the interfacial SEI layer, which typically acts as an ionic transport bottleneck and contributes to interfacial resistance, can be significantly improved through moderate O doping. However, excessive O incorporation reduces the bulk SSE conductivity and destabilizes the SEI phase thermodynamically, thereby increasing the likelihood of impurity formation. Therefore, an optimal O‐doping level that balances enhanced ionic conductivity with phase stability is expected to be most desirable. A quantitative comparison with experimental results relevant to the optimal O‐doping level is provided in the Discussion section.

## 6 Electronic Passivation and Kinetic Stabilization at the SEI | SSE Interface

One of the key requirements for a passivating SEI is the suppression of further interfacial reactions by inhibiting electron transport from the Li metal, thereby rendering the interface kinetically stable. To achieve this, the SEI phase should be electronically insulating, preventing electrons from reaching the electrolyte and driving further chemical reductions. Therefore, a finite bandgap is a useful indicator of electronic passivation. We selected Li_2_S_0.75_P_0.125_Cl_0.125_ and Li_2_S_0.688_P_0.125_Cl_0.125_O_0.062_ as model SEI phases for band‐gap calculations, as they are compositionally similar to the expected SEI compositions (Li_2_S_0.72_P_0.14_Cl_0.14_ for the undoped case and Li_2_S_0.68_P_0.14_Cl_0.14_O_0.04_ for the O‐doped case). Notably, the O‐doped model SEI phase contains a higher oxygen content than the expected composition. This choice provides a conservative assessment of electronic passivation, given that, in the present phase, oxygen incorporation is associated with a reduced bandgap. DFT calculations using the HSE06 hybrid functional confirmed that both undoped and O‐doped phases are semiconducting, with finite bandgaps of 2.7 and 2.68 eV, respectively (Section S12). The possible influence of structural disorder and Li‐vacancy defects was further examined in Section S12. These additional analyses indicate that the examined disordered and defective model structures do not exhibit a finite DOS at the Fermi level. Nevertheless, the present calculations do not explicitly quantify electron tunneling, thermally activated hopping between localized defect states [30], defect‐assisted transport at higher defect concentrations, or extended defects such as grain boundaries [31, 32, 33]. These effects may further influence electronic passivation and should be investigated in future studies.

**FIGURE 5:**
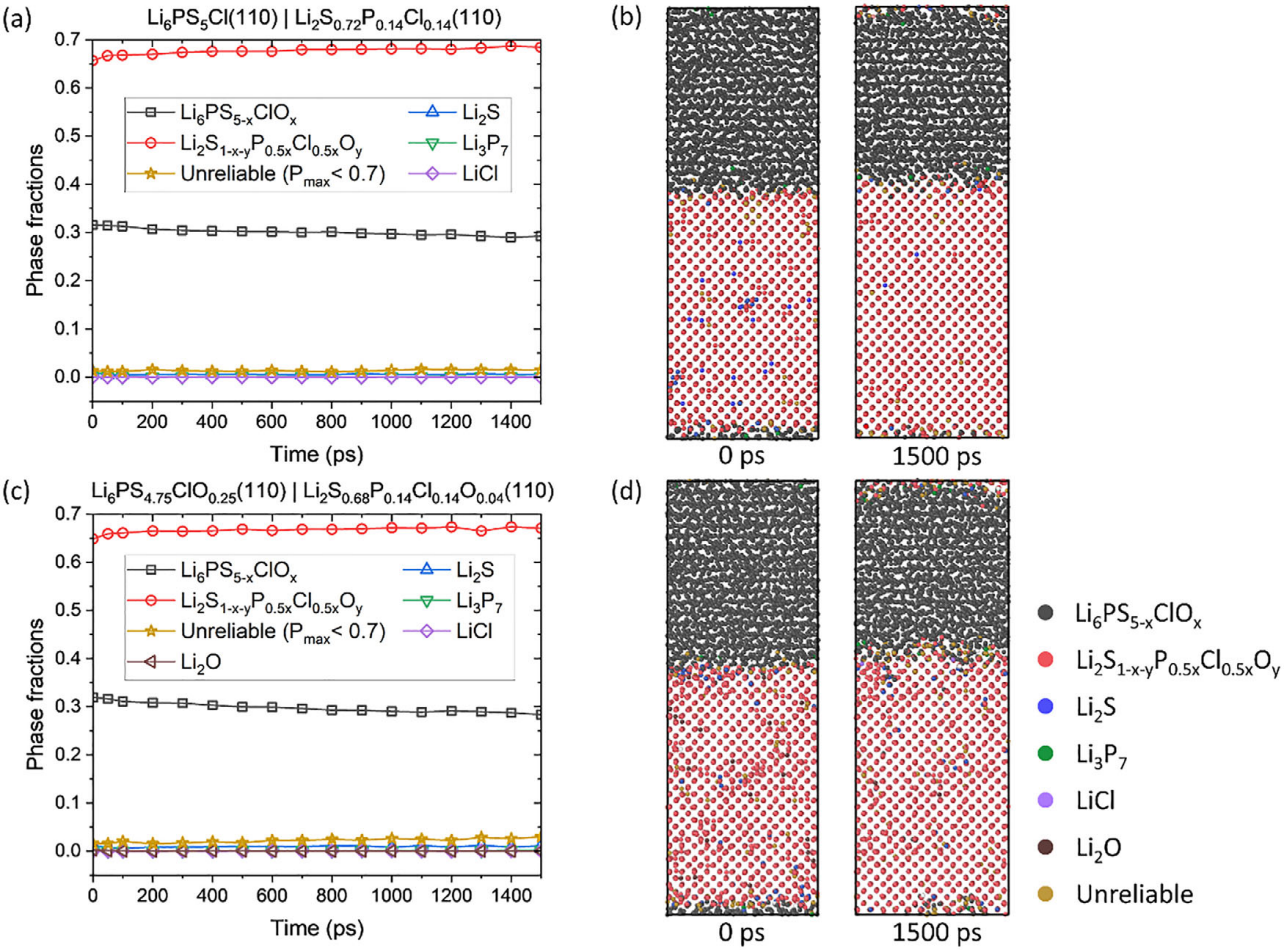
Time evolution of phase fractions for (a) Li_6_PS_5_Cl(110) | Li_2_S_0.72_P_0.14_Cl_0.14_(110) and (c) Li_6_PS_4.75_ClO_0.25_(110) | Li_2_S_0.68_P_0.14_Cl_0.14_O_0.04_(110) interfaces at 600 K under NPT conditions (1000 bar), following 100 ps of equilibration. (b, d) Atom‐wise phase labels for Li atoms at 0 and 1500 ps for the (b) Li_6_PS_5_Cl(110) | Li_2_S_0.72_P_0.14_Cl_0.14_(110) and (d) Li_6_PS_4.75_ClO_0.25_(110) | Li_2_S_0.68_P_0.14_Cl_0.14_O_0.04_(110) interfaces, respectively. Only Li atoms are shown in (b) and (d), and colors indicate the phases assigned by the ML‐based phase identification model.

Assuming that a sufficiently thick SEI has already formed, we further assessed its passivating nature by constructing undoped and O‐doped SEI | Li_6_PS_5‐x_ClO_x_ interface models and simulating them at 600 K for 1.5 ns to evaluate interfacial reactivity. As shown in Figure 5, applying the ML‐based phase identification model to trajectory snapshots indicates that, under both undoped and O‐doped conditions, the Li_6_PS_5‐x_ClO_x_ region remains largely intact. In contrast to the rapid interfacial reactions observed in the direct Li | Li_6_PS_5‐x_ClO_x_ interface simulations under identical conditions, these results suggest kinetic stabilization at the SEI | Li_6_PS_5‐x_ClO_x_ interface. Furthermore, phonon calculations confirmed that both Li_2_S_0.75_P_0.125_Cl_0.125_ and Li_2_S_0.625_P_0.125_Cl_0.125_O_0.125_ are dynamically stable phases (Section S13).

## 7 Discussions

### 7.1 Validation and Plausibility of the Dominant SEI Phase

**FIGURE 6:**
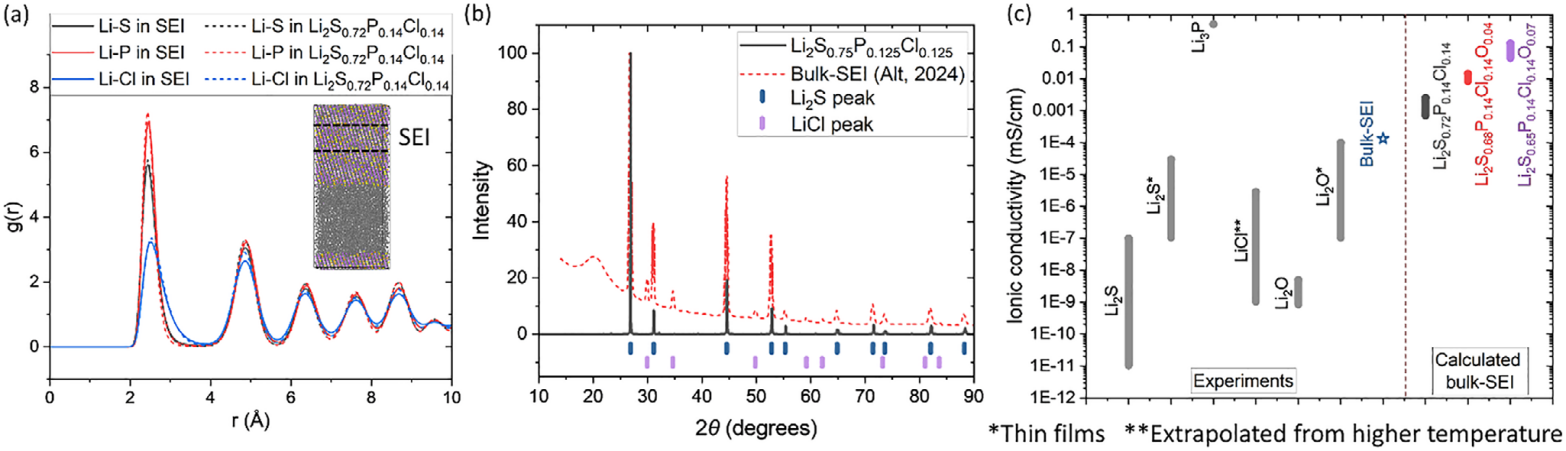
(a) RDF comparison at 600 K between the SEI phase extracted from the MD‐derived interfacial structure and the bulk‐SEI phase (Li_2_S_0.72_P_0.14_Cl_0.14_). (b) Simulated XRD pattern of Li_2_S_0.75_P_0.125_Cl_0.125_ (black solid line). The DFT‐optimized structure obtained with the optB88‐vdW functional was used to compute the XRD pattern. The reference XRD pattern (red dashed line) of the bulk‐SEI and the Bragg peak positions of Li_2_S (blue) and LiCl (purple) were digitized from Alt et al. [34]. The XRD pattern was intensity‐normalized for comparison. (c) Experimental ranges of ionic conductivity at 300 K for Li_2_S [36], thin‐film Li_2_S (Li_2_S*) [37], Li_3_P [38], LiCl [39, 40], Li_2_O [41], thin‐film Li_2_O (Li_2_O*) [37], and bulk‐SEI [34]. The calculated ionic conductivity of bulk‐SEI is also shown for comparison.

From our MLIP‐MD simulations combined with the ML‐based phase identification model, the dominant SEI phase formed at the undoped SSE | Li interface was identified as Li_2_S_1‐x_P_0.5x_Cl_0.5x_. To validate the plausibility of this phase identification, we first compare its local structural characteristics with those directly obtained from the interface MD simulations. Figure 6a shows the RDFs calculated at 600 K for bulk Li_2_S_1‐x_P_0.5x_Cl_0.5x_ and for the SEI region formed in the Li_6_PS_5_Cl(110) | Li(110) interface MD simulation (hereafter, ‘the MD‐derived SEI’) at the same temperature. To preserve the S:P:Cl ratio of Li_6_PS_5_Cl, the bulk‐SEI composition was parameterized as Li_2_S_0.72_P_0.14_Cl_0.14_. The RDF of the MD‐derived SEI was computed by selecting Li atoms within the interfacial region (110 Å < z < 130 Å) at 500 ps, a time at which sufficient crystallization of the SEI had occurred. The close agreement between the RDFs of bulk Li_2_S_0.72_P_0.14_Cl_0.14_ and the MD‐derived SEI indicates that the Li_2_S_0.72_P_0.14_Cl_0.14_ phase captures the essential structural motifs of the interfacial SEI. The structural similarity between the MD‐derived SEI and the bulk‐SEI phase is further examined through a coordination number analysis in Section S8.

Next, we compare our predictions with available experimental observations. Direct experimental characterization of SEI microstructure is inherently challenging due to the buried nature of the interface. To overcome this limitation, Alt et al. [34] synthesized a bulk‐phase SEI via direct reaction between Li_6_PS_5_Cl and Li metal powder and characterized its microstructural and transport properties. X‐ray diffraction (XRD) analysis of the synthesized bulk‐SEI identified Li_2_S as the dominant phase (89 wt.%), with LiCl accounting for the remaining 11 wt.% (noting that the Li_6_PS_5_Cl precursor already contained ∼3 wt.% LiCl as an impurity). Because Li_2_S_0.72_P_0.14_Cl_0.14_ shares the same underlying crystal structure as Li_2_S, its XRD peak positions are expected to closely resemble those of Li_2_S. Indeed, as shown in Figure 6b, the XRD peaks calculated from a DFT‐optimized Li_2_S_0.75_P_0.125_Cl_0.125_ structure closely match the experimentally measured XRD pattern of the bulk‐SEI sample. Furthermore, EDX mapping results presented by Alt et al. [34] show that, although some regions exhibit local enrichment of S, P, and Cl, these elements are largely distributed throughout the SEI. Considering that Li_3_P is not clearly resolved in XRD while P is observed to be broadly distributed in elemental mapping, and that Cl may exhibit partial inhomogeneity due to residual LiCl impurity from the precursor, these observations are consistent with a dominant Li_2_S_1‐x_P_0.5x_Cl_0.5x_‐ type phase rather than discrete stoichiometric decomposition products (Li_2_S, LiCl, and Li_3_P). Moreover, Schwietert et al. [13] demonstrated that the experimentally observed electrochemical stability window of Li_6_PS_5_Cl (∼1.25 V) is much wider than the thermodynamically predicted window (∼0.3 V). This finding indicates that interfacial decomposition is not governed solely by the thermodynamic stability of the final decomposition products (Li_2_S, LiCl, and Li_3_P), but is instead controlled by kinetically accessible and metastable phases.

Taken together, this dominant SEI Li_2_S_1‐x_P_0.5x_Cl_0.5x_‐type phase is expected to play a central role in Li‐ion transport at the Li | SSE interface. Accordingly, we constructed bulk Li_2_S_0.72_P_0.14_Cl_0.14_ structures, along with O‐doped structures Li_2_S_0.68_P_0.14_Cl_0.14_O_0.04_ and Li_2_S_0.65_P_0.14_Cl_0.14_O_0.07_, to evaluate their ionic conductivities at 300 K (see Methods Section). As shown in Figure 6c, the ionic conductivity of Li_2_S_0.72_P_0.14_Cl_0.14_ is within one order of magnitude of experimentally reported bulk‐SEI conductivities. This discrepancy falls within the typical range often observed between simulation and experimental measurements. In contrast, the experimentally measured bulk‐SEI ionic conductivity differs substantially from that of pristine Li_2_S, the thermodynamically expected dominant decomposition product, which typically exhibits a much lower ionic conductivity (∼10^−8^ mS/cm) [35]. Upon O‐doping, the ionic conductivity further increases with increasing O content, reinforcing the role of structurally disordered, O‐containing Li_2_S‐based phases in facilitating Li‐ion transport across the interface.

### 7.2 Experimental Observations on O‐Doped SSE | Li Metal Interfaces

Building on the atomistic insights obtained in this work, we now connect our findings to available experimental studies on O‐doped argyrodite solid electrolytes and their interfaces with Li metal. As discussed in Section 5, increasing the O‐doping level enhances the ionic conductivity of the SEI; however, this improvement comes at the expense of a slight decrease in the bulk SSE conductivity and a reduced thermodynamic stability of the SEI phase, which can promote impurity formation. This trade‐off implies that, rather than maximizing the oxygen content, an optimal and moderate level of O doping is critical for balancing interfacial transport gains against penalties in bulk SSE transport and SEI phase stability.

This interpretation is supported by several experimental studies. Wu et al. reported that an O‐doped Li_6_PS_5_Cl composition (Li_6.05_PS_4.9_O_0.1_Cl_1.05_) in LiCoO_2_|Li_6.05_PS_4.9_O_0.1_Cl_1.05_|Li cells exhibits a much lower interfacial charge‐transfer resistance after 100 cycles compared with the undoped Li_6_PS_5_Cl electrolyte [42]. Although we did not explicitly evaluate how O doping affects the SEI thickness in this study, our results suggest that the enhanced ionic conductivity of the SEI induced by O doping can contribute to the experimentally observed reduction in interfacial resistance. More recently, Ming et al. demonstrated that O‐doped argyrodite electrolytes improve interfacial stability in Li | Li_5.5_PS_4.5_Cl_1.5_ | Li symmetric cells [43]. Specifically, the O‐doped composition Li_5.5_PS_4.425_Cl_1.5_O_0.075_ exhibited a higher critical current density, prolonged stable cycling, and lower interfacial resistance after 100 cycles compared with the undoped electrolyte. These observations are consistent with our finding that O doping enhances Li‐ion transport across the SEI.

The importance of an appropriate O‐doping level is further highlighted by the work of Indrawan et al. [23], who systematically investigated Li_6_PS_5‐2.5x_O_2.5x_Cl (x = 0, 0.05, 0.1) using Li | SSE | Li symmetric cells under DC polarization at 0.1 mA cm^−2^. They found that a moderate oxygen content (x = 0.05) prolongs the Li‐metal lifetime, enabling stable cycling for 197 cycles, approximately 5.5 times longer than that of the undoped argyrodite (x = 0). In contrast, a higher oxygen content (x = 0.1) resulted in only intermediate stability of 52 cycles, indicating inferior performance than the optimally doped composition.

Taken together, these experimental observations underscore a non‐monotonic dependence of the interfacial performance on the O‐doping level. This behavior can be rationalized by our atomistic findings, which elucidate the competing mechanisms: while moderate O substitution facilitates interfacial Li‐ion transport, excessive O doping imposes a trade‐off by compromising both the thermodynamic stability of the SEI and the bulk ionic conductivity of the SSE.

### 7.3 Estimation of SEI‐induced Resistance and Effective Ionic Conductivity

To relate the calculated ionic conductivities to interfacial transport, we estimated the SEI‐induced area‐specific ionic resistance by treating the SSE and the SEI layer as two ion‐conducting layers connected in series along the through‐plane transport direction. In this approximation, the SEI resistance is given by *R_SEI_* = *d_SEI_* /σ_*SEI*_, where *σ_SEI_* denotes the Li ion conductivity of the SEI phase. Here, we approximate the effective SEI conductivity relevant to the ionic resistance as *σ_SEI ∼_ σ_ion_*, assuming that the ionic conductivity dominates over the electronic conductivity, i.e., *σ_ion >>_ σ_el,_*, where *σ_el_* is the electronic conductivity [34]. Thus, the calculated *R_SEI_* should be interpreted as the Li ion conduction resistance of the SEI layer, rather than the full electrochemical interfacial impedance. The total area‐specific ionic resistance is expressed as

(2) $$R_{total}=\frac{L_{SSE}}{\sigma_{SSE}}+\frac{d_{SEI}}{\sigma_{SEI}}$$

where *L_SSE_* is the thickness of the SSE, *d_SEI_* is the SEI thickness, and *σ_SSE_* and *σ_SEI_* are the ionic conductivities of the SSE and SEI, respectively. The ionic conductivity values used in this analysis are those shown in Figure 4a,b. The corresponding effective ionic conductivity of the combined SSE/SEI stack was then obtained from

(3) $$\sigma_{eff}=\frac{L_{SSE}+d_{SEI}}{R_{total}}\cdot$$

**FIGURE 7:**
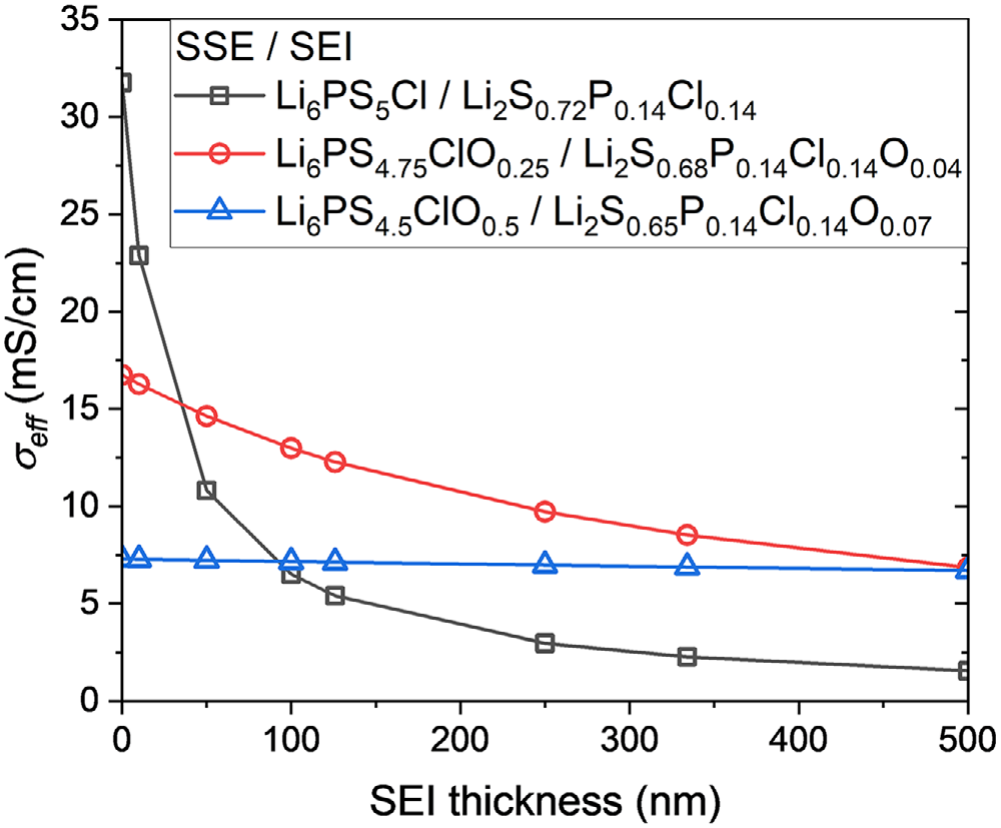
Estimated effective ionic conductivity of the SSE/SEI stack as a function of SEI thickness. The SSE thickness was fixed at *L_SSE_* = 500 µm. The legend denotes each SSE and its corresponding SEI composition formed upon reaction with Li metal, represented as an SSE/SEI stack.

Figure 7 shows the calculated effective ionic conductivity as a function of SEI thickness for the undoped and O‐doped systems, using *L_SSE_* = 500 µm. In the absence of an SEI layer, Li_6_PS_5_Cl exhibits the highest ionic conductivity among the three systems. However, its effective ionic conductivity decreases rapidly as the SEI thickness increases because the Li_6_PS_5_Cl‐derived SEI has the lowest Li ion conductivity. In contrast, the O‐doped systems show a much weaker decrease in *σ_eff_* with increasing SEI thickness, owing to the enhanced Li ion conductivity of their corresponding SEI phases, as shown in Figure 4a,b. In particular, the Li_6_PS_4.75_ClO_0.25_ / Li_2_S_0.68_P_0.14_Cl_0.14_O_0.04_ (SSE / SEI) stack provides the highest *σ_eff_* over a broad SEI thickness range because it retains relatively high bulk SSE conductivity while substantially improving the SEI conductivity. The Li_6_PS_4.5_ClO_0.5_ / Li_2_S_0.65_P_0.14_Cl_0.14_O_0.07_ (SSE / SEI) shows the smallest sensitivity to SEI growth, reflecting the highest SEI conductivity, although its lower bulk SSE conductivity limits the absolute *σ_eff_* at small SEI thickness.

This analysis demonstrates that the effective ionic transport across the Li | SSE interface cannot be inferred solely from the bulk SSE conductivity. Although Li_6_PS_5_Cl has the highest bulk ionic conductivity, the low ionic conductivity of its SEI can dominate the total ionic resistance once an interphase layer forms. In contrast, oxygen doping slightly reduces the bulk SSE conductivity but significantly enhances the Li ion conductivity of the resulting SEI, thereby mitigating the SEI‐induced resistance increase. This trade‐off explains why moderate oxygen incorporation can be beneficial for effective interfacial ion transport, even when the bulk SSE conductivity is partially sacrificed. The full thickness‐dependent values of *R_SEI_*, *R_total_*, and *σ_eff_* are provided in Section S14.

## 8 Conclusions

In this study, we combined MLIP‐MD simulations with a ML‐based phase identification framework to investigate kinetically formed SEI phases at the Li_6_PS_5‐x_ClO_x_ | Li interface and to clarify how O doping influences both bulk and interfacial properties. By explicitly introducing O doping in the interface simulations and directly analyzing the resulting SEI structures, we disentangled the competing roles of oxygen incorporation in the bulk electrolyte and in the interphase. Our results reveal a clear trade‐off: increasing the O‐doping level enhances ionic transport within the SEI, leading to higher SEI ionic conductivity, whereas the bulk ionic conductivity of the argyrodite electrolyte decreases with increasing O content. Moreover, while O doping improves SEI transport, it simultaneously reduces the thermodynamic stability of the SEI phases, thereby increasing the likelihood of impurity formation. This atomistic perspective provides a consistent explanation for the experimentally observed non‐monotonic dependence on the O‐doping level.

The integration of MLIP‐MD with a machine‐learning‐based phase identification model offers a general and transferable approach to probe buried solid‐solid interfaces that are difficult to characterize experimentally. This framework can be extended to assess the effects of diverse dopants and various solid electrolytes, providing a practical route to rationally design interfaces that otherwise act as ionic transport bottlenecks in all‐solid‐state batteries.

## 9 Methods

### 9.1 MLIP Construction

In MLIP training using SevenNet, the cutoff radius of 5 Å was used to define local atomic environments, and spherical harmonics were included up to *l_max_* = 2. The network consisted of three interaction layers, with each layer using 32 channels, providing a practical balance between model expressiveness and computational efficiency. Train and validation set was divided as 9:1 ratio. Details of the training dataset and the effect of training set composition on the interfacial MD simulations are described in Sections S1 and S2.

### 9.2 DFT Calculation

All DFT calculations were performed using the projector augmented‐wave (PAW) method [44, 45] as implemented in Vienna ab‐initio simulation package (VASP) [46, 47, 48]. Ab initio molecular dynamics (AIMD) simulations were first conducted to efficiently sample atomic configurations, after which accurate energies and forces were obtained from static single‐point calculations.

For the AIMD simulations, the Perdew–Burke–Ernzerhof (PBE) exchange‐correlation functional [49] was employed. A plane‐wave energy cutoff of 400 eV and Γ – point sampling of the Brillouin zone were used, with a timestep of 2 fs. Electronic self‐consistency was achieved with an energy convergence criterion of 10^−4^ eV.

To obtain high‐accuracy energies and forces for the configurations sampled from AIMD, static single‐point calculations were performed under more rigorous settings. The plane‐wave cutoff was increased to 520 eV, and the optB88‐vdw functional [50] was employed. The Brillouin‐zone was sampled using a Γ – centered k‐point mesh generated by the fully automatic scheme in VASP with a length parameter of 40. In this scheme, the number of subdivisions along each reciprocal lattice vector **b** *_i_* is determined by *N_i_* =  int(*R_k_* |**b**_*i*_| + 0.5), where *R_k_* indicates the length parameter that controls the k‐point density. The electronic SCF convergence threshold was set to 10^−6^ eV. Spin‐polarization was employed only for the isolated two‐atom in vacuum configurations used to represent short‐range two‐body interactions, while all other training configurations were computed in the non‐spin‐polarized mode.

### 9.3 MLIP‐MD Simulation

Molecular dynamics (MD) simulations were performed using LAMMPS code [51] with the constructed SevenNet‐based MLIP. A timestep of 1 fs was employed. Each system was first relaxed and then heated from 300 K to the target temperature (300, 400, and 600 K) over 100 ps under the NVT ensemble using a Nosé–Hoover thermostat. After equilibration, the production run was carried out at the target temperature in the NPT ensemble using a Nosé‐Hoover thermostat and barostat [52, 53] at a pressure of 1000 bar. The production run time was 2 ns for the Li_6_PS_5‐x_ClO_x_ | Li interface systems and 1 ns for bulk crystal systems, including Li_6_PS_5‐x_ClO_x_ and Li_2_S_1‐x‐y_P_0.5x_Cl_0.5x_O_y_ phases. Unless otherwise noted, the reported simulation times refer to the production runs and exclude the equilibration time.

Interface structures for the Li_6_PS_5‐x_ClO_x_ | Li systems were generated using the CoherentInterfaceBuilder implemented in the pymatgen [54] package. The Li_6_PS_5‐x_ClO_x_ | Li interface supercells contained 17 024 atoms. For Li_6_PS_5‐x_ClO_x_(110) | Li_2_S_0.72‐x_P_0.14_Cl_0.14_O_x_(110) interfaces, supercells of 12 924 atoms were used.

Bulk Li_6_PS_5‐x_ClO_x_ models for diffusion coefficient calculations were built using a 4 × 4 × 4 supercell of Li_6_PS_5_Cl (3328 atoms). The anti‐site defect structures were generated by exchanging S anions on the 4d sites with Cl anions on the 4a sites in Li_6_PS_5_Cl. In the 52‐atom unit cell, there are four S atoms at the 4d sites and four Cl atoms at the 4a sites. We enumerated all distinct anti‐site structures corresponding to anti‐site defect ratios *R_as_* = 0, 0.25, 0.5, 0.75, and 1.0, and selected the lowest‐energy structure for each *R_as_*. For O‐doped structures, O substitution was introduced starting from the lowest‐energy anti‐site structure identified at each *R_as_* (i.e., the corresponding O‐free structure). Specifically, O was substituted for S atoms occupying the 16e sites by considering all possible substitution patterns for a given O content (x = 0.25 and 0.5 in Li_6_PS_5‐x_ClO_x_). All generated structures were fully relaxed using our MLIP, and their energies were compared after relaxation. The most stable structure at each *R_as_* and O doping level was then used to compute the diffusion coefficient.

For Li_2_S_0.72‐x_P_0.14_Cl_0.14_O_x_ bulk diffusion coefficient calculations, we constructed a Li_2_S supercell containing 2592 atoms as a 6 × 6 × 6 replication of the 12‐atom Li_2_S unit cell (equivalently, 6 × 6 × 3 of the 24‐atom 1 × 1 × 2 Li_2_S unit cell). Substitutional P and Cl dopants were introduced on the anion sublattice by replacing S atoms, while all Li atoms were kept fixed in number. In each 1 × 1 × 2 Li_2_S unit cell within the supercell, two S atoms were first replaced by one P and one Cl atom, ensuring a homogenous baseline distribution of P and Cl throughout the structure. To achieve the target composition of Li_2_S_0.72_P_0.14_Cl_0.14_, additional P‐Cl substitution pairs were introduced in a subset of the unit cells: one additional S atom was replaced by P, and a neighboring S atom closest to this P site was replaced by Cl. For each composition, 300 different structures were generated by randomly sampling the substitution sites. All generated structures were subsequently used for structural relaxation using our MLIP. From this ensemble, five randomly selected relaxed structures were used for the diffusion coefficient calculations. To construct O‐doped SEI models based on these five selected structures, additional O substitutions were introduced on S sites. To avoid artificial dopant clustering, the simulation cell was partitioned into subdomains, and one S atom was randomly selected from each subdomain for O substitution. Additional S atoms were then randomly selected from the remaining S sites to reach the target O doping level.

### 9.4 Reduction Fraction of Solid Electrolytes

Upon contact with Li metal, the Li_6_PS_5_Cl electrolyte undergoes reduction, characterized by the sequential breaking of P─S bonds. During the breaking of P─S bonds, the oxidation state of P changes from +5 in PS_4_ units to ‐3 in elemental P [15]. The reduction fraction can be defined as [15]

(4) $$f_{r}=\frac{8N\left(P\right)+6N\left(PS\right)+4N\left(PS2\right)+2N\left(PS3\right)}{8Z}$$

where *N(PS_x_)* is the number of phosphorus atoms coordinated by *x* anions, and *Z* is the total number of electrolyte formula units. The coordination number was determined by counting the number of anion neighbors (S or O) within a 3 Å cutoff around each P atom. In O‐doped systems, both P─S and P─O bonds were treated equivalently when evaluating the local coordination environment of phosphorus.

### 9.5 Ionic Conductivity Calculations

The mean square displacement (MSD) over a time interval *Δt* was obtained by averaging the squared displacements over all possible time origins *t* that satisfy *t* + *Δt* ≤ *t_tot_*, as well as over all mobile ions, according to

(5) $$\mathrm{MSD}\left(\Delta t\right)=\frac{1}{N}\sum_{i=1}^{N}{\langle {\left|r_{i}\left(t+\Delta t\right)-r_{i}\left(t\right)\right|}^{2}\rangle }_{t}$$

where *t_tot_* is the total length of the MD trajectory, *N* is the number of mobile ions, and *i* indexes individual Li ions. The notation 〈···〉_*t*_ denotes averaging over all possible time origins *t* for a given *Δt*. The diffusion coefficient *D* was then obtained from the Einstein relation,

(6) $$D=\frac{MSD\left(\Delta t\right)}{2d\Delta t}$$

where *d* is the dimensionality of the system (*d* = 3). The statistical uncertainty of *D* was estimated based on the total effective number of diffusion events sampled during the simulation, following the approach proposed by He et al. [55]. Both *D* and its statistical uncertainty were calculated using the aimd.diffusion module [55].

Ionic conductivity was calculated using the Nernst–Einstein equation. Diffusion coefficients were first obtained from simulations at 600, 700, and 800 K, fitted to an Arrhenius relationship, and then extrapolated to 300 K to evaluate the ionic conductivity at room temperature. The corresponding Arrhenius plots for Li_2_S_0.72‐x_P_0.14_Cl_0.14_O_x_ are provided in Section S11.

### 9.6 Machine‐Learning‐Based Phase Identification Model

We trained a multi‐layer perceptron (MLP) classifier implemented in scikit‐learn [56] to predict class labels from input feature vectors. The input feature matrix **X** was constructed from Li‐centered atomic node embeddings extracted from the last hidden layer of the NequIP‐based SevenNet model, and phase labels were integer‐encoded as y. The dataset was split into training, validation, and test sets using stratified random sampling with a ratio of 8:1:1. All input features were standardized using z‐score normalization, with normalization parameters fitted on the training set and then applied to the validation and test sets. The MLP classifier consisted of two fully connected hidden layers with 32 units each and ReLU activation functions. The model achieving the highest validation macro‐F1 score during training was selected; this selected model achieved a test set macro‐F1 score of 0.9972. The learning curves of the macro‐F1 scores for the training and validation sets, as well as details of the training set for the phase identification model, are provided in Section S6.

## Author Contributions

**Sojeong Yang**: conceptualization, methodology, software, data curation, investigation, validation, formal analysis, visualization, Writing – original draft. **Jung‐Hoon Lee**: resources, funding acquisition, project administration. **Byungju Lee**: conceptualization, methodology, investigation, funding acquisition, project administration, visualization, supervision, resources, writing – review and editing. **Hyun‐Jae Lee**: formal analysis. **Sungwoo Kang**: methodology. **Atefeh Yadegarifard**: formal analysis. **Nongnuch Artrith**: writing – review and editing.

## Funding

This work was supported by the Nano & Material Technology Development Program through the National Research Foundation of Korea (NRF) funded by the Ministry of Science and ICT (RS‐2024‐00407995, 50%), National Supercomputing Center with supercomputing resources including technical support (KSC‐2024‐CRE‐0576, 25%), and the KU‐KIST Graduate School of Converging Science and Technology (KU‐KIST school program, 25%).

## Conflicts of Interest

The authors declare no conflicts of interest.

## Supporting information




**Supporting File**: advs76563‐sup‐0001‐SuppMat.pdf.

## Data Availability

The data that supports the findings of this study are available in the supplementary material of this article.
