# Supplementary material for: Enhanced Ionic Conductivity at the Solid Electrolyte Interphase of Oxygen‐Doped Li6PS5Cl
Source: Adv Sci (Weinh). 2026 Jul 13:e76563. Online ahead of print. doi: 10.1002/advs.76563 (PMC13360120; doi:10.1002/advs.76563)
Supplement: Supplementary file 1 — Supporting File: advs76563‐sup‐0001‐SuppMat.pdf. [file ADVS-9999-e76563-s001.pdf]

Supporting Information

Enhanced ionic conductivity at the solid electrolyte interphase of oxygen-doped Li_6PS_5Cl

Sojeong Yang [a], Sungwoo Kang [a], Atefeh Yadegarifard [a,b], Hyun-Jae Lee [a], Nongnuch Artrith [c], Jung-Hoon Lee [a,d], Byungju Lee [a,b,]*

<Contents>

A. MLIP training and test dataset

B. Effect of excluding decomposition products from the MLIP training set

C. Interfacial reactions of Li_6PS_5Cl and $Li_6PS_4ClO_1$ with Li metal

D. Diffusion behavior of P, S, and Cl at the $Li_6PS_{5-x}ClO_x$(110) | Li(110) interface

E. Extended MD simulations at 300 and 400 K

F. Training set for the machine-learning-based phase identification model

G. ML-based phase identification model without $Li_2S_{1-x}P_{0.5x}Cl_{0.5x}$ phase in the training set

H. Structural correspondence between the MD-derived SEI and the proposed bulk-SEI phase

I. Ionic conductivity and thermodynamic stability of $Li_6PS_{5-x}ClO_x$ as a function of anti-site defect ratio

J. Microscopic origin of enhanced Li-ion conductivity in O-doped bulk-SEI phases

K. Arrhenius plots of Li-ion diffusion coefficient

L. Electronic density of states of bulk-SEI phase

M. Structural relaxation and phonon calculations

N. Thickness-dependent resistance analysis of the SSE/SEI stack

A. MLIP training and test dataset

The training dataset for the amorphous and bulk Li_6PS_5Cl phases was adopted from the work of Lee et al.,[1] which was generated using identical DFT settings for energy and force calculations. For the bulk phase, we further augmented the dataset by curating structures from the SevenNet-0 training dataset by selecting Li-P-S-Cl-O compounds. These structures underwent structural optimizations, and the initial and final structures from each optimization trajectory were included in the training dataset. In addition, we incorporated possible decomposition phases, including (i) experimentally observed and thermodynamically stable compounds in the Materials Project database (E_{hull} < 20 meV/atom) and (ii) decomposition products predicted under interfacial reaction conditions (Table S3 and S4). Structures for these phases were sampled via AIMD, followed by single-point self-consistent field (SCF) calculations to obtain reference energies and forces. To better capture short-range repulsion at small interatomic distances, we also included structures constructed to represent two-body short-range interactions. All details of the training dataset are summarized in Table S1.

The test dataset was sampled from AIMD simulations of the Li_6PS_5Cl(110) | Li(110) interface at 1000 K, using a larger interfacial supercell (142 atoms) than those included in the training dataset. The RMSEs of the predicted energies and forces on the test dataset are 4.2 meV/atom and 0.094 eV/Å, respectively. A comparison of DFT reference values and MLIP predictions for the energies and forces in the test dataset is shown in Figure S1. The training dataset and trained MLIP model are available at https://github.com/KIST-CSRC/MLIP-LPSCO-Li-interface/.

Table S1. Data types, structures, and number of structures in the MLIP training dataset. The total dataset comprises 8981 structures, including datasets adopted from Lee et al.[1]

Data type	Structures	Number of structures (temperature)
Interface	Li_6PS_5Cl(100) | Li(100)	50 (300K→600K), 50 (300K→1000K), 300 (600K), 347 (1000K)
	Li_6PS_5Cl(110) | Li(110)	50 (300K→600K), 50 (300K→1000K), 300 (600K), 300 (1000K)
	$Li_6PS_{4.75}O_{0.25}$(110) | Li(110)	50 (600K), 50 (1000K)
	$Li_6PS_{4.75}O_{0.25}$(100) | Li(100)	50 (600K), 50 (1000K)
	$Li_6PS_{4.5}ClO_{0.5}$(100) | Li(100)	50 (300K→600K), 50 (300K→1000K), 250 (600K), 300 (1000K)
	$Li_6PS_{4.5}ClO_{0.5}$(110) | Li(110)	50 (300K→600K), 50 (300K→1000K), 250 (600K), 250 (1000K)
	Li_2O(100) | Li(100)	50 (300K→600K), 50 (300K→1000K), 50 (600K), 50 (1000K)
	Li_2S(100) | Li(100)	50 (300K→600K), 50 (300K→1000K), 50 (600K), 50 (1000K)
	Li_3P(100) | Li(100)	50 (300K→600K), 50 (300K→1000K), 50 (600K), 50 (1000K)
	LiCl(100) | Li(100)	50 (300K→600K), 50 (300K→1000K), 50 (600K), 50 (1000K)
Surface	Li(100)	6 (0 K), 20 (600K), 60 (1000K)
	Li(110)	6 (0 K), 60 (1000K)
	Li_6PS_5Cl(100)	6 (0 K), 60 (600K)

	Li_6PS_5Cl(110)	6 (0 K)
	$Li_6PS_{4.75}O_{0.25}$(100)	12 (0K)
	$Li_6PS_{4.75}O_{0.25}$(110)	12 (0K)
Bulk	Li_6PS_5Cl	1001 (Lee et al.[1]; 700K ~ 1000K), 6 (0K)
	Li	3 (0K), 100 (300K), 125 (600K), 125 (1000K)
	$Li_6PS_{4.75}ClO_{0.25}$	250 (600K), 250 (1000K)
	$Li_6PS_{4.5}ClO_{0.5}$	100 (1000K)
	Li_2O_2	50 (300K)
	$Li_2S_2O_7$	25 (300K)
	Li_2S	25 (300K), 50 (600K), 21 (1000K)
	Li_2SO_4	50 (300K)
	Li_3PO_4	25 (300K), 50 (600K)
	$Li_4P_2O_7$	75 (300K)
	$Li_7P_3S_{11}$	25 (300K)
	$LiClO_4$	25 (300K)
	LiP_5	25 (300K)
	$LiPO_3$	75 (300K)
	Li_3PS_4	50 (600K)
	Li_3ClO	50 (600K)
	Li_3P_7	50 (600K)
	LiCl	50 (600K), 21 (1000K)
	LiP_7	50 (1000K)
	LiP	50 (600K)
	Li_2O	50 (600K), 21 (1000K)
	P	50 (600K)
	Li_3P	50 (600K), 21 (1000K)
	From the SevenNet-0 training set (including Li, P, S, Cl, and O)	240 (0K; oxygen-only structures excluded)
**Amorphous**	Li_6PS_5Cl	1675 (Lee et al.[1]; 2000K, 2000K→300K, 500K)
**Two-body**	Li, P, S, Cl, O combinations	127 (0K)
**Total**		**8981**

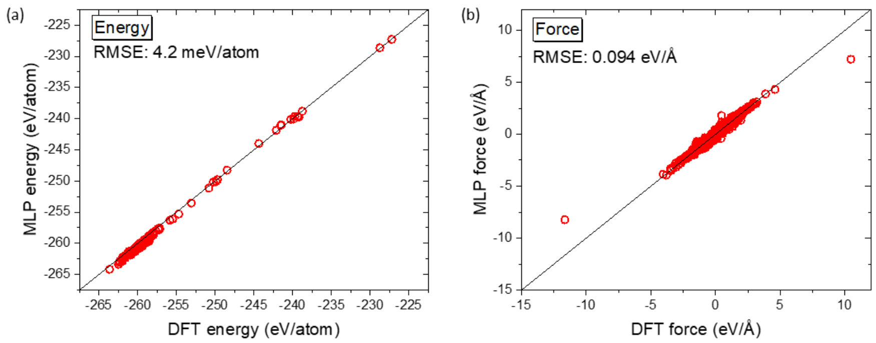

Figure S1. Comparison of (a) energies and (b) forces for the test dataset calculated using DFT and MLIP constructed in this study.

B. Effect of excluding decomposition products from the MLIP training set

To assess whether the predicted SEI structure depends on the explicit inclusion of thermodynamically expected decomposition products in the training set, we trained an additional MLIP, denoted *MLIP-reduced*, using the same training protocol as *MLIP-full* but excluding all structures associated with the predicted decomposition phases (Li_3P, LiCl, Li_2S, and Li_2O) from the training data. The training set for *MLIP-reduced* is listed in Table S2. The train/validation split was maintained at 9:1, yielding RMSEs of 7.96 meV/atom for energy and 0.125 eV/Å for forces on the training set, and 7.57 meV/atom and 0.127 eV/Å, respectively, on the validation set.

Using *MLIP-reduced*, we performed MD simulations at 600 K starting from the same initial Li_6PS_5Cl(110) | Li(110) interface structure as used in the main text. The resulting Li ion MSD profile, shown in Figure S2(a), exhibits three distinct diffusion regimes (fast, moderate, and slow), consistent with the behavior described in Section 3 of the main text. The transition from the fast to the moderate diffusion regime occurs at approximately 30 ps, coinciding with the point at which the reduction fraction reaches unity (Figure S2(b)), followed by a second transition to the slow diffusion regime at approximately 300 ps. Visualization of the interface structure at 200 ps confirms partial crystallization during the moderate diffusion regime, whereas nearly complete crystallization is observed at 500 and 2000 ps during the slow diffusion regime, as shown in Figure S3. A slightly steeper MSD slope in *MLIP-reduced* than in *MLIP-full* is attributed to a larger fraction of phase boundaries between the crystallized domains, which act as additional fast Li-ion conduction pathways. Furthermore, phase identification using the trained ML model (Figure S2(c)) identifies $Li_2S_{1-x-y}P_{0.5x}Cl_{0.5x}O_y$ as the dominant SEI phase in the *MLIP-reduced* MD simulation, in close agreement with the results obtained using the original MLIP (*MLIP-full*). These results demonstrate that the predicted interfacial structure and SEI phase are robust against the exclusion of decomposition-related phases from the training set, confirming that the predicted SEI formation is not an artifact of pre-including the thermodynamically expected decomposition products in the training set.

Table S2. Training set for *MLIP-reduced*, which excludes predicted decomposition products (Li_3P, LiCl, Li_2S, Li_2O).

Data type	Structures	Number of structures (temperature)
**Interface**	Li_6PS_5Cl(100) | Li(100)	50 (300K→600K), 50 (300K→1000K), 300 (600K), 347 (1000K)
	Li_6PS_5Cl(110) | Li(110)	50 (300K→600K), 50 (300K→1000K), 300 (600K), 300 (1000K)
	$Li_6PS_{4.75}O_{0.25}$(110) | Li(110)	50 (600K), 50 (1000K)

	$Li_6PS_{4.75}O_{0.25}(100)$ | Li(100)	50 (600K), 50 (1000K)
	$Li_6PS_{4.5}ClO_{0.5}(100)$ | Li(100)	50 (300K→600K), 50 (300K→1000K), 250 (600K), 300 (1000K)
	$Li_6PS_{4.5}ClO_{0.5}(110)$ | Li(110)	50 (300K→600K), 50 (300K→1000K), 250 (600K), 250 (1000K)
**Surface**	Li(100)	6 (0 K), 20 (600K), 60 (1000K)
	Li(110)	6 (0 K), 60 (1000K)
	$Li_6PS_5Cl(100)$	6 (0 K), 60 (600K)
	$Li_6PS_5Cl(110)$	6 (0 K)
	$Li_6PS_{4.75}O_{0.25}(100)$	12 (0K)
	$Li_6PS_{4.75}O_{0.25}(110)$	12 (0K)
**Bulk**	Li_6PS_5Cl	1001 (Lee et al.[1]; 700K ~ 1000K), 6 (0K)
	Li	3 (0K), 125 (600K), 125 (1000K)
	$Li_6PS_{4.75}ClO_{0.25}$	250 (600K), 250 (1000K)
	$Li_6PS_{4.5}ClO_{0.5}$	100 (1000K)
**Amorphous**	Li_6PS_5Cl	1675 (Lee et al.[1]; 2000K, 2000K→300K, 500K)
**Two-body**	Li, P, S, Cl, O combinations	127 (0K)
**Total**		**6807**

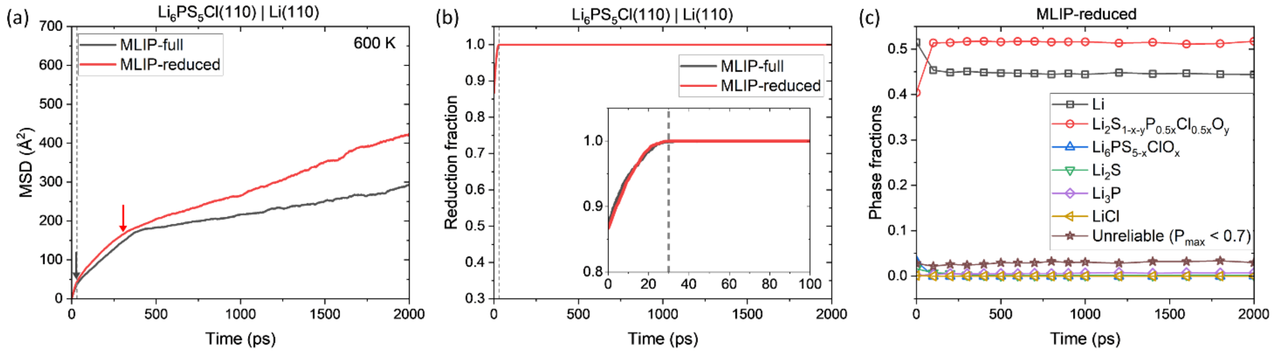

Figure S2. MLIP-MD simulations of $Li_6PS_5Cl(110)$ | Li(110) interface at 600 K using *MLIP-full* and *MLIP-reduced*. (a) MSD of Li ions as a function of simulation time. Transition points from the fast to moderate and from the moderate to slow diffusion regimes are indicated by black and red arrows, respectively. (b) Reduction fraction of Li_6PS_5Cl as a function of time. The grey dashed line indicates the point at which the reduction fraction reaches unity, corresponding to the fast-to-moderate diffusion regime transition in (a). (c) Time evolution of the phase fractions predicted by the phase identification ML model.

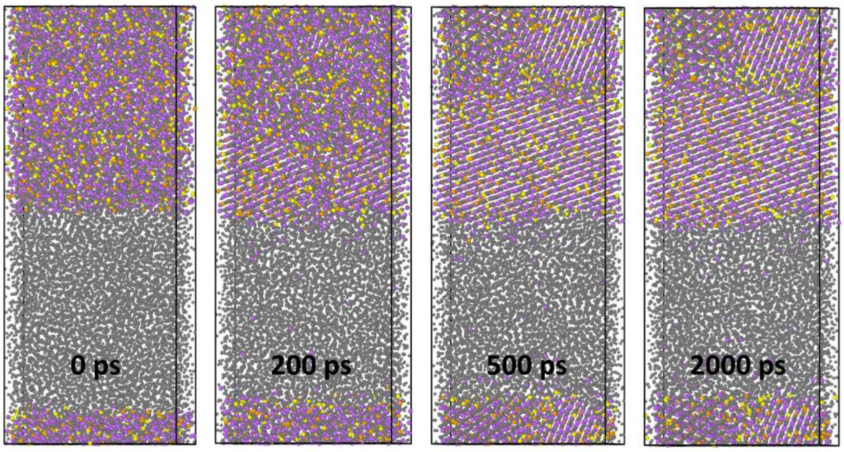

Figure S3. Atomic snapshots of the $Li_6PS_5Cl(110)$ | Li(110) interface at representative simulation times (200, 500, and 2000 ps) from *MLIP-reduced* MD simulations at 600 K under the NPT ensemble at 1000 bar.

C. Interfacial reactions of Li_6PS_5Cl and $Li_6PS_4ClO_1$ with Li metal

Table S3. Decomposition products and reaction energies for interfacial reactions between Li_6PS_5Cl and Li metal, calculated using our MLIP: xLi + (1-x)Li_6PS_5Cl.

x	Reaction	Reaction energy (eV/atom)
0	Li_6PS_5Cl → Li_3PS_4 + Li_2S + LiCl	-0.08881
0.278	0.8333 Li + 0.1667 Li_6PS_5Cl → 0.8333 Li_2S + 0.1667 LiCl + 0.1667 P	-0.59888
0.283	0.8372 Li + 0.1628 Li_6PS_5Cl → 0.02326 LiP_7 + 0.814 Li_2S + 0.1628 LiCl	-0.60689
0.295	0.8444 Li + 0.1556 Li_6PS_5Cl → 0.02222 Li_3P_7 + 0.7778 Li_2S + 0.1556 LiCl	-0.61693
0.316	0.8571 Li + 0.1429 Li_6PS_5Cl → 0.1429 LiP + 0.7143 Li_2S + 0.1429 LiCl	-0.63072
0.381	0.8889 Li + 0.1111 Li_6PS_5Cl → 0.1111 Li_3P + 0.5556 Li_2S + 0.1111 LiCl	-0.66483
1	Li → Li	0

Table S4. Decomposition products and reaction energies for interfacial reactions between $Li_6PS_4ClO_1$ and Li metal, calculated using our MLIP: xLi + (1-x)$Li_6PS_4ClO_1$.

x	Reaction	Reaction energy (eV/atom)
0	Li_6PS_4ClO → 0.75 Li_3PS_4 + Li_2S + 0.25 Li_3PO_4 + LiCl	-0.04877
0.224	0.7895 Li + 0.2105 Li_6PS_4ClO → 0.8421 Li2S + 0.05263 Li_3PO_4 + 0.2105 LiCl + 0.1579 P	-0.46953
0.229	0.7941 Li + 0.2059 Li_6PS_4ClO → 0.02206 LiP_7 + 0.8235 Li_2S + 0.05147 Li_3PO_4 + 0.2059 LiCl	-0.4758

0.238	0.8028 Li + 0.1972 Li_6PS_4ClO → 0.02113 Li_3P_7 + 0.7887 Li_2S + 0.0493 Li_3PO_4 + 0.1972 LiCl	-0.48615
0.257	0.8182 Li + 0.1818 Li_6PS_4ClO → 0.1364 LiP + 0.7273 Li_2S + 0.04545 Li_3PO_4 + 0.1818 LiCl	-0.50106
0.316	0.8571 Li + 0.1429 Li_6PS_4ClO → 0.1071 Li_3P + 0.5714 Li_2S + 0.03571 Li_3PO_4 + 0.1429 LiCl	-0.53766
0.381	0.8889 Li + 0.1111 Li_6PS_4ClO → 0.1111 Li_3P + 0.4444 Li_2S + 0.1111Li_2O + 0.1111 LiCl	-0.57158
1	Li → Li	0

D. Diffusion behavior of P, S, and Cl at the $Li_6PS_{5-x}ClO_x$(110) | Li(110) interface

Figure S4 presents the mean squared displacement (MSD) of P, S, and Cl atoms at the Li_6PS_5Cl(110) | Li(110) interface. As illustrated in the initial structure following the equilibration run (Figure S4(a)), the MSD was calculated for P, S, and Cl atoms located within the 100 Å < z < 130 Å region, with the results shown in Figure S4(b). For the Li_6PS_5Cl(110) | Li(110) interface, these atoms become nearly immobile even at 600 K once they enter the slow diffusion regime (t > 400 ps), which signifies the formation of a fully crystallized SEI.

The diffusion characteristics of the O-doped $Li_6PS_{4.75}ClO_{0.25}$(110) | Li(110) interface are shown in Figure S5(a). A sharp decrease in the slope of the MSD for P, S, and Cl is observed at approximately 1050 ps, indicating a transition into the slow diffusion regime. Similar to the undoped system, atoms at the O-doped interface remain largely immobile in this regime, with the minor displacements arising mainly from grain boundary formation within the newly formed SEI, as shown in Figure S5(b).

Overall, the onset of the abrupt decrease in MSD slope is consistent with the initiation of the slow diffusion regime discussed in Section 3 of the main text. The immobility of P, S, and Cl suggests that the phase separation into distinct phases, such as Li_3P and LiCl, is kinetically unfavorable. Furthermore, snapshots reveal a sparse distribution of P, S, and Cl atoms rather than dense clusters, providing further evidence that the formation of $Li_2S_{1-x}P_{0.5x}Cl_{0.5x}$ phase is more plausible that the separation into individual binary phases.

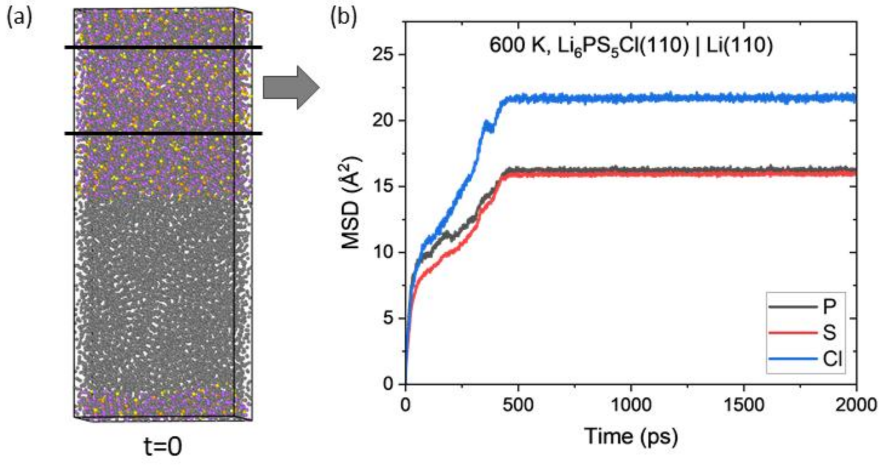

Figure S4. (a) Snapshot of the initial structure of the production run after equilibration from 300 K to 600 K. Atoms located within the region defined by the solid lines (100 Å < z < 130 Å) were selected for MSD calculations to minimize the influence of interface-specific kinetics associated with the Li metal. (b) MSD of the selected P, S, and Cl atoms in Li_6PS_5Cl(110) | Li(110) at 600 K.

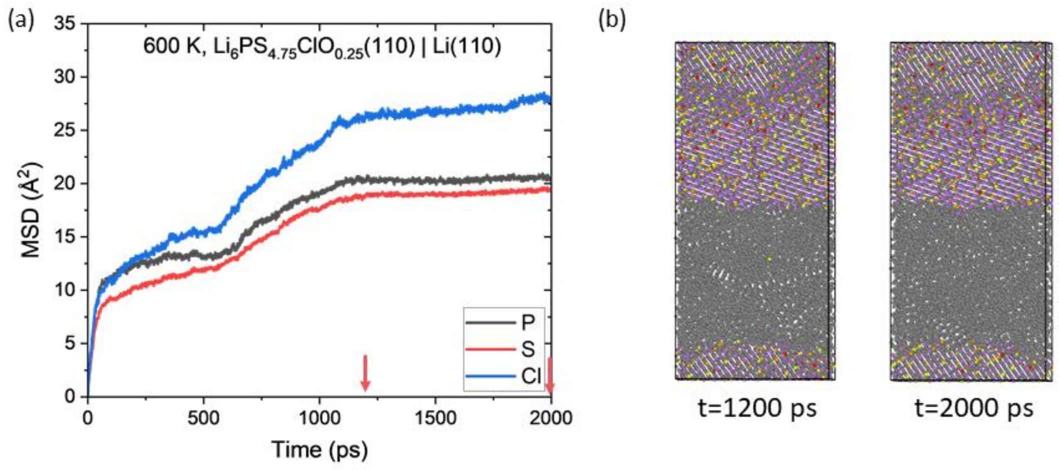

Figure S5. (a) MSD of P, S, and Cl at the $Li_6PS_{4.75}ClO_{0.25}$(110) | Li(110) interface at 600 K. (b) Snapshots of interfacial structures at 1200 and 2000 ps, corresponding to the red arrows in (a).

E. Extended MD simulations at 300 and 400 K

To examine whether the three diffusion regimes observed at 600 K are also reproduced under lower-temperature conditions, we performed extended MD simulations at 300 and 400 K for 8 ns. The results in Figure S6(a) show that the transition from the fast to the moderate diffusion regime occurs at approximately 400 ps at 400 K and approximately 2900 ps at 300 K. These transition points are in good agreement with the time at which the reduction fraction of the Li_6PS_5Cl electrolyte reaches unity, as shown in Figure S6(b). A similar correspondence between the fast-to-moderate diffusion transition and the point at which the calculated reduction fraction reaches unity was also observed at 600 K. This indicates that the initial rapid Li ion motion is closely associated with the reduction of the electrolyte. The subsequent transition from the moderate to the slow diffusion regime is observed at approximately 6800 ps at 400 K, whereas no clear transition to the slow diffusion regime is detected within 8 ns at 300 K. To further understand the structural evolution at 400 K, we examined the atomic structures at 4000 and 8000 ps. At 4000 ps, corresponding to the moderate diffusion regime, crystallization is still in progress, and a partially crystallized structure is observed, as shown in Figure S6(c). At 8000 ps, corresponding to the slow diffusion regime, the interfacial region is nearly fully crystallized.

We further analyzed the time evolution of the phase fractions predicted by the classifier at 300 and 400 K, as shown in Figure S7. At both temperatures, the dominant SEI phase is identified as $Li_2S_{1-x-y}P_{0.5x}Cl_{0.5x}O_y$, consistent with the phase evolution observed at 600 K. The main difference is that the formation of this dominant SEI phase is delayed at lower temperatures, reflecting slower interfacial reaction and crystallization kinetics.

These results demonstrate that the lower-temperature simulations reproduce the same MSD-regime behavior and dominant SEI phase evolution observed at 600 K, with the regime transitions and SEI formation primarily shifted to longer timescales due to slower kinetics. This consistency indicates that the diffusion-regime behavior observed at 600 K reflects the same underlying interfacial evolution that occurs at lower temperatures, but on an accelerated timescale. It also supports the relevance of the SEI phase identified at 600 K to lower temperature conditions, as the classifier predicts the same dominant SEI phase, $Li_2S_{1-x-y}P_{0.5x}Cl_{0.5x}O_y$, at both 300 and 400 K despite the slower formation kinetics. Therefore, the 600 K simulation can be regarded as an accelerated representation of the same interfacial reaction and crystallization pathway, rather than as a condition that produces fundamentally different SEI chemistry. Although complete SEI crystallization at 300 K may occur on a timescale longer than that accessible in the present MD simulations, the crystallized SEI phase identified

at 600 K is consistent with the lower temperature phase evolution and can serve as a reasonable representative model for the dominant SEI formed through Li_6PS_5Cl reduction at the Li metal interface.

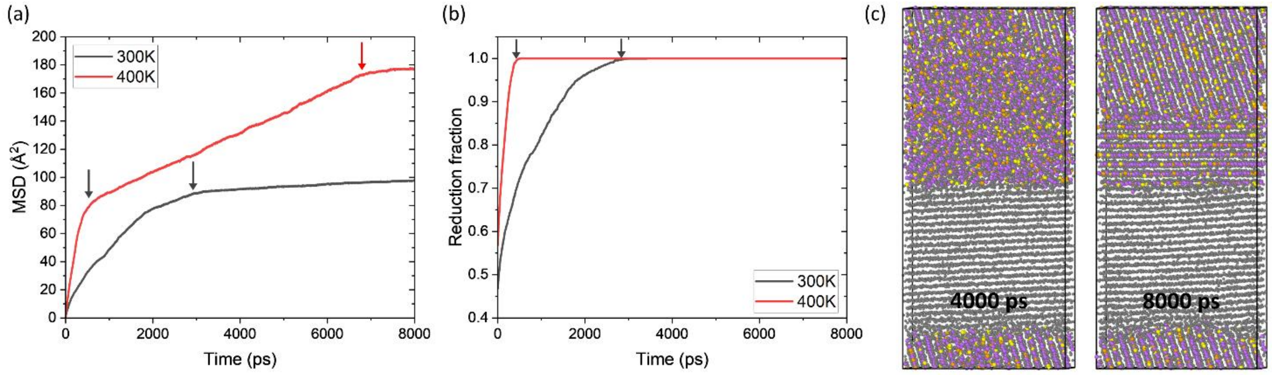

Figure S6. (a) MSD of Li ions at the $Li_6PS_5Cl(110)$ | Li(110) interface from 8 ns MD simulations at 300 and 400 K under the NPT ensemble at 1000 bar. The transition points from the fast to the moderate diffusion regime and from the moderate to the slow diffusion regime are marked by black and red arrows, respectively. (b) Reduction fraction of the Li_6PS_5Cl electrolyte during MD simulations at 300 and 400 K. (c) Atomic snapshots of the interface at 400 K after 4000 and 8000 ps.

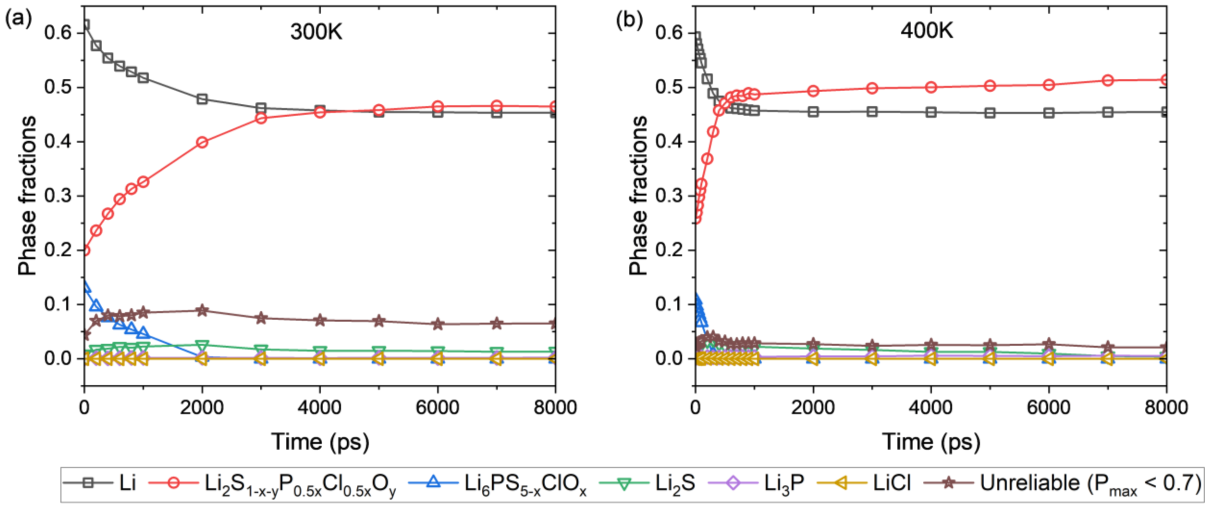

Figure S7. Time evolution of the phase fractions predicted by the classifier during 8 ns MD simulations of the $Li_6PS_5Cl(110)$ | Li(110) interface with R_{as}=0, performed under the NPT ensemble at 1000 bar and at (a) 300 K and (b) 400 K.

F. Training set for the machine-learning-based phase identification model

A multi-layer perceptron (MLP) classifier was trained using reference phases selected from the Materials Project database according to the following criteria (the full training set is listed in Table S5): (1) Li-containing compounds composed of two or more elements among Li, P, S, Cl, and O; (2) experimentally observed structures; (3) thermodynamic stability with an energy above hull E_{hull} < 20 meV/atom; and (4) for compositions with multiple stable polymorphs, only one representative structure was included. In addition to these reference phases, Li (bcc), the solid electrolyte phase ($Li_6PS_{5-x}ClO_x$), and possible SEI phase ($Li_2S_{1-x-y}P_{0.5x}Cl_{0.5x}O_y$) were also included in the training set. For each phase, multiple snapshots sampled from AIMD simulations were used to construct the training set, thereby accounting for finite-temperature structural fluctuations. The learning curves of the training and validation macro-F1 scores are shown in Figure S8.

To qualitatively assess how the phase-dependent local environments are represented in the learned embedding space, we visualize the Li atom embeddings of the training phases using t-SNE[2] in Figure S9. Perplexity, an effective number of neighbors, is set as 40. The resulting projection shows that Li atoms from different phases tend to form distinct clusters, indicating that the embeddings capture phase-specific local chemical environments.

Table S5. Phases used for the ML-based phase identification model, AIMD simulation temperatures, and the number of embeddings (corresponding to the number of Li atoms).

	**Phases**	**Temperature (K)**	**Number of embeddings (Li atoms)**
0	$Li_6PS_{5-x}ClO_x$ (x = 0, 0.25, 0.5)	0, 300, 600	2976
1	Li_2O_2	300	900
2	Li_2O	600	3200
3	$Li_2S_2O_7$	300	200
4	Li_2SO_4	300	400
5	$Li_2S_{1-x-y}P_{0.5x}Cl_{0.5x}O_y$<br>(x, y)=(0.25, 0), (0.25, 0.125),<br>(0.28, 0), (0.28, 0.04), (0.28, 0.07)	0, 300	8576
6	Li_2S	600	3200
7	Li (bcc)	600	6750
8	Li_3P_7	600	1200
9	Li_3PO_4	600	2400
10	Li_3P	600	2400
11	$Li_4P_2O_7$	300	400
12	$Li_7P_3S_{11}$	300	700
13	$LiClO_4$	300	200
14	LiCl	600	1600
15	LiP_5	300	400
16	LiP_7	300	400
17	$LiPO_3$	300	300
18	LiP	600	1600
**Total**			**37802**

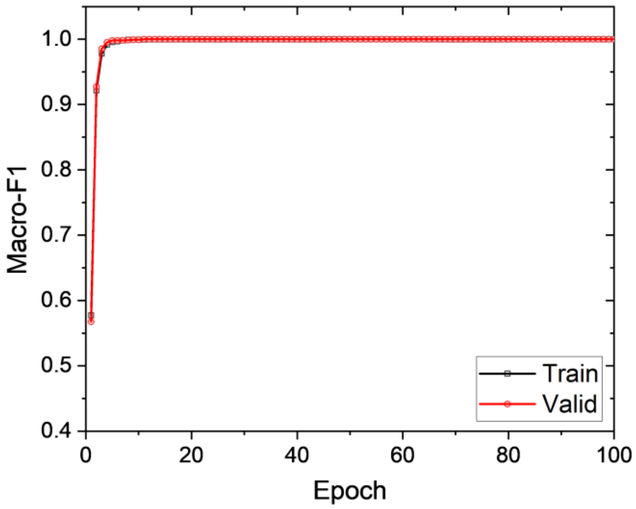

Figure S8. Learning curves of the macro-F1 scores for the training (black) and validation (red) sets as a function of training epoch for the ML-based phase identification model, showing convergence of model performance during training.

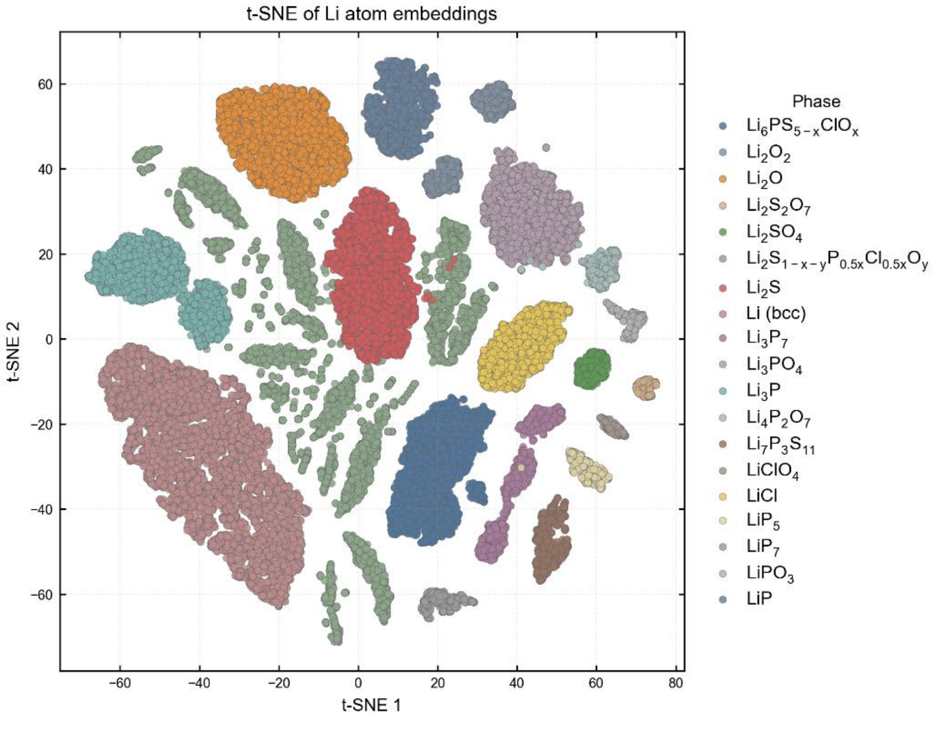

Figure S9. t-SNE visualization of Li atom embeddings for the crystalline phases included in the training dataset of the ML-based phase identification model. Each point represents a Li atom, colored by phase. Li atoms associated with different phases tend to cluster in distinct regions of the embedding space.

G. ML-based phase identification model without $Li_2S_{1-x}P_{0.5x}Cl_{0.5x}$ phase in the training set

Figure S10 shows the time evolution of phase fractions predicted along the MD simulation trajectory by a phase identification model trained on a reduced training set that excludes the $Li_2S_{1-x}P_{0.5x}Cl_{0.5x}$ phase. This reduced dataset is hereafter referred to as *Dataset-1*, while the full training set listed in Table S5, which includes the $Li_2S_{1-x}P_{0.5x}Cl_{0.5x}$ phase, is referred to as *Dataset-2*. The predictions are shown for the $Li_6PS_5Cl(110)$ | Li(110) interfacial system, simulated at 600 K, with an anti-site defect ratio R_{as} = 0 in the argyrodite phase. Using the model trained on *Dataset-1*, the majority of SEI formed during the MD simulation are identified as Li_2S. In addition, a small fraction of Li atoms are assigned to unreliable phases, defined by cases where the maximum class probability does not exceed 0.7. It should be noted that the MLP classification model assigns class probabilities based on relative scores learned in the embedding space. As a result, even if the embedding vector of a given Li atom is substantially dissimilar to all reference phases in an absolute sense, a comparatively high maximum probability may still be assigned when the embedding lies marginally closer to the decision region associated with a particular phase (e.g. Li_2S) than to those of other reference phases. This motivates an examination of whether the reference phases used for training adequately cover the embedding space of Li atoms in the MD-derived SEI during interfacial MD simulations.

To this end, t-SNE was employed as a visualization tool to assess the representativeness of the training datasets. t-SNE enables a qualitative comparison of local atomic environments by preserving neighborhood relationships in the high-dimensional embedding space. The t-SNE embedding was constructed using both the *Dataset-1* and the target embeddings, where the target embeddings correspond to Li atom embeddings extracted from the SEI region of the $Li_6PS_5Cl(110)$ | Li(110) structure at the final timestep (t = 2000 ps) of the MD simulation at 600 K. A perplexity of 40 was used for all t-SNE analyses.

Figure S11(a,b) shows the t-SNE visualizations based on a joint embedding constructed from *Dataset-1* and the target embeddings, with the target embeddings omitted in Figure S11(a) to improve visual clarity. From this comparison, it is observed that the target embeddings occupy regions of the low-dimensional space that are distinct from those associated with the phases included in *Dataset-1*, indicating that the local atomic environments of the SEI formed during the interfacial MD simulation may not be fully represented by *Dataset-1*.

In contrast, Figure S12(a,b) shows the corresponding t-SNE results obtained using *Dataset-2*, which includes the $Li_2S_{1-x}P_{0.5x}Cl_{0.5x}$ phase. As before, Figure S12(a) presents the visualization of *Dataset-2* alone, while Figure S12(b) includes both *Dataset-2* and the target embeddings. In this case, the target embeddings appear in close proximity to the $Li_2S_{1-x}P_{0.5x}Cl_{0.5x}$ phase in the low-dimensional space, suggesting a strong similarity in local atomic environments. It should be noted that the $Li_2S_{1-x}P_{0.5x}Cl_{0.5x}$ phase can exhibit a wide range of local Li environments depending on the substitution sites and spatial distributions of P and Cl within the Li_2S framework. Because the training set does not exhaustively sample all possible substitutional configurations, some target embeddings may not be fully contained within the region occupied by the $Li_2S_{1-x}P_{0.5x}Cl_{0.5x}$ phase in the t-SNE visualization.

Taken together, these results indicate that the inclusion of the $Li_2S_{1-x}P_{0.5x}Cl_{0.5x}$ phase in the training dataset is essential for adequately capturing the local atomic environments present in the SEI formed during interfacial MD simulations. This observation provides a clear justification for the construction and use of *Dataset-2* in this work.

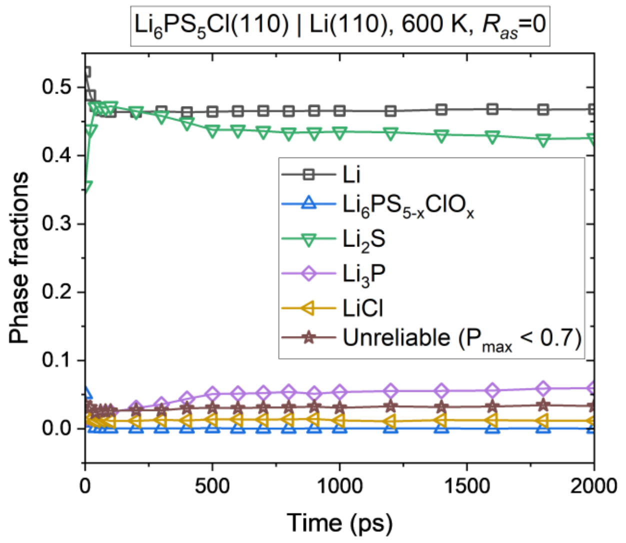

Figure S10. Time evolution of phase fractions predicted by the phase identification model trained on *Dataset-1*, which excludes the $Li_2S_{1-x}P_{0.5x}Cl_{0.5x}$ phase from the original training dataset. The interfacial system used for this prediction is identical to that shown in Figure 3(b) of the main text.

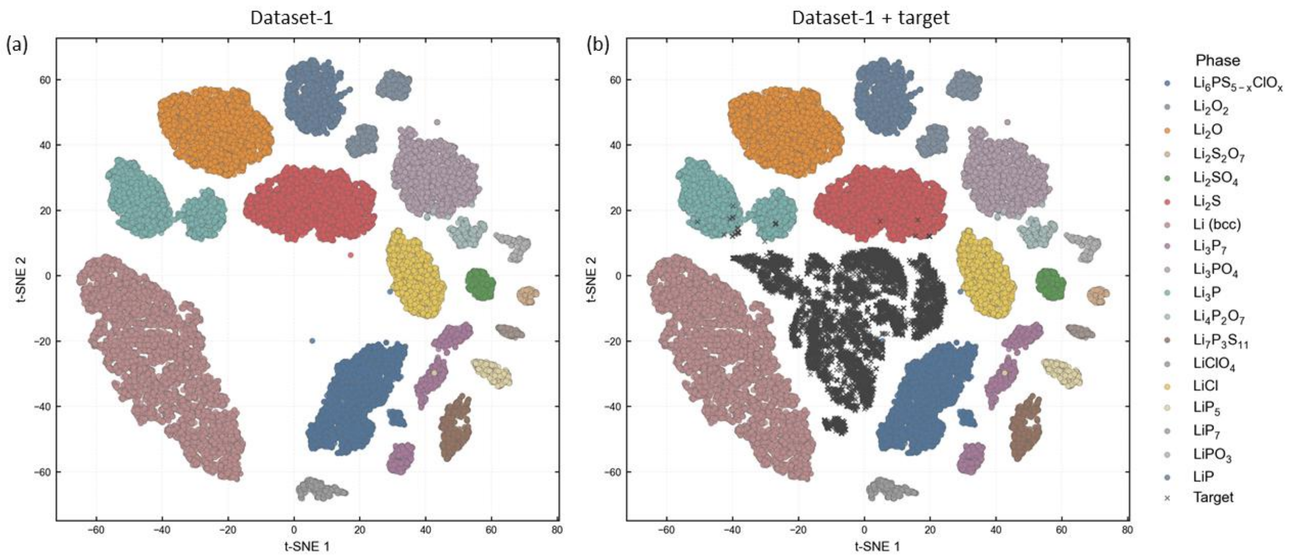

Figure S11. t-SNE visualization based on a joint embedding constructed using *Dataset-1* and target embeddings. Panel (a) shows *Dataset-1* alone, with the target embeddings omitted for visual clarity, while panel (b) includes both *Dataset-1* and the target embeddings.

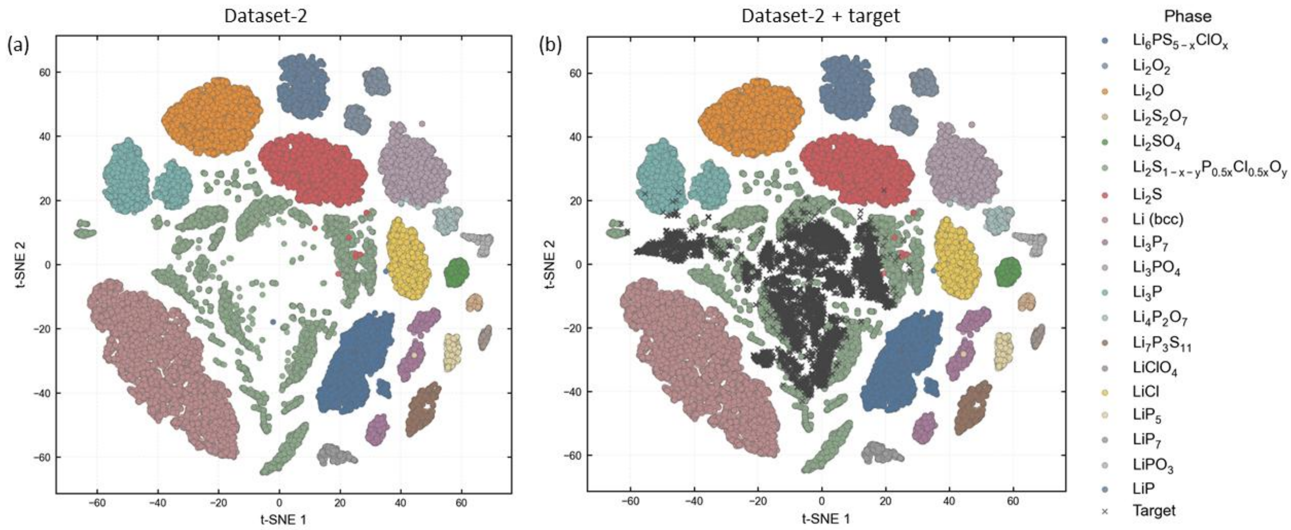

Figure S12. t-SNE visualization based on a joint embedding constructed using *Dataset-2* and target embeddings. Panel (a) shows *Dataset-2* alone, with the target embeddings omitted for visual clarity, while panel (b) includes both *Dataset-2* and the target embeddings.

H. Structural correspondence between the MD-derived SEI and the proposed bulk-SEI phase

In Figure 6(a) of the main text, we compared the RDFs of the SEI phase extracted from the MD-derived interfacial structure with those of the proposed bulk-SEI phase, $Li_2S_{0.72}P_{0.14}Cl_{0.14}$. To further assess whether the MD-derived SEI is better described by the proposed bulk-SEI phase or by conventional decomposition products, we additionally compared its RDFs with those of Li_2S, Li_3P, and LiCl, as shown in Figure S13. The RDF of the MD-derived SEI shows close agreement with that of the bulk $Li_2S_{0.72}P_{0.14}Cl_{0.14}$ phase, whereas noticeable differences are observed when compared with Li_2S, Li_3P, and LiCl, not only in peak positions but also in peak intensities and first-shell features. These differences indicate that the proposed bulk SEI phase provides a more representative description of the MD-derived SEI structure than the conventional decomposition products.

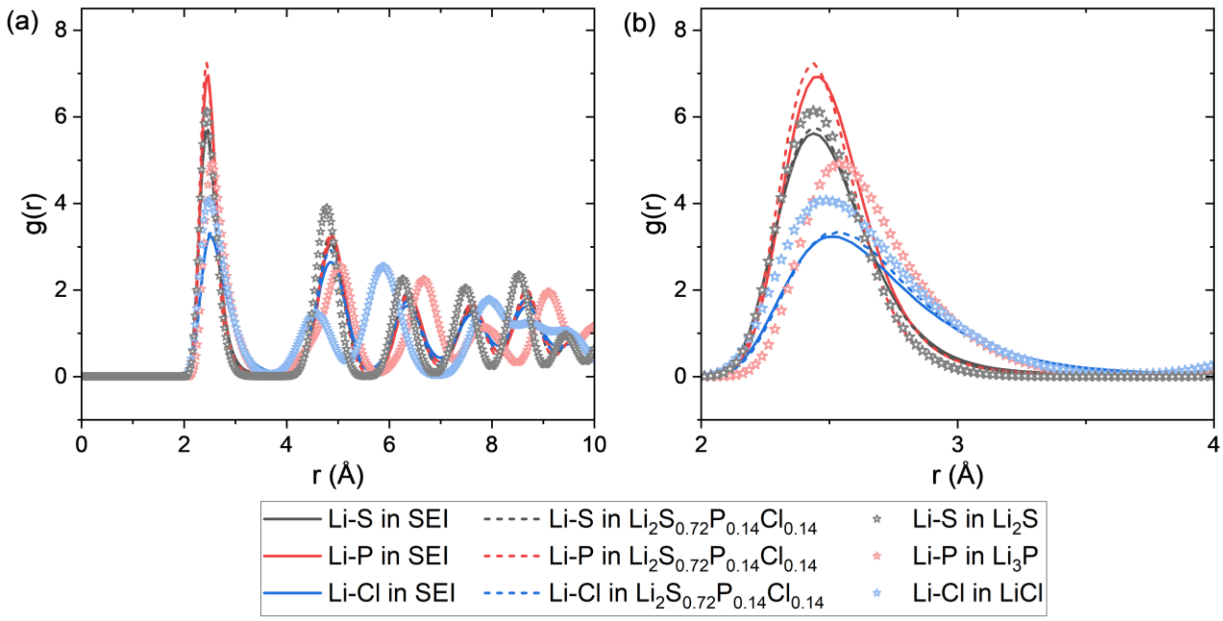

Figure S13. (a) RDF comparison at 600 K between the SEI phase extracted from the MD-derived interfacial structure, the bulk-SEI phase ($Li_2S_{0.72}P_{0.14}Cl_{0.14}$), and the conventional decomposition products Li_2S, Li_3P, and LiCl. (b) Enlarged view of the first-shell region, highlighting the local structural similarity among the compared phases.

To further examine whether the MD-derived SEI can be represented by the proposed bulk SEI phase, $Li_2S_{0.72}P_{0.14}Cl_{0.14}$, we compared their local coordination environments using time- and space-resolved coordination number (CN) analysis, as shown in Figure S14. Overall, the CN distributions are relatively uniform across the SEI region, except near the Li metal side. This indicates that the MD-derived SEI develops a relatively homogeneous local coordination environment over the SEI region, rather than separating into strongly segregated Li_2S-, LiCl-, or Li_3P-rich domains.

To quantify this behavior, we averaged the coordination numbers over the SEI region defined by 85 Å < z < 140 Å and plotted their time evolution in Figure S15 for the Li-P, Li-Cl, and Li-S pairs. The corresponding CN values of the proposed bulk-SEI phase, $Li_2S_{0.72}P_{0.14}Cl_{0.14}$, are shown for comparison, together with the reference CN values of conventional decomposition products, namely Li-P in Li_3P, Li-Cl in LiCl, and Li-S in Li_2S. The resulting CNs gradually approach the corresponding values of the proposed bulk-SEI phase, indicating that the local coordination environment of the MD-derived SEI becomes consistent with that of the proposed bulk-SEI phase. This consistency, together with the relatively uniform CN distributions along the z-coordinate shown in Figure S14, provides classifier-independent structural evidence supporting the assignment of the

dominant SEI as a $Li_2S_{1-x}P_{0.5x}Cl_{0.5x}$-type phase, rather than a physical mixture of conventional decomposition products such as Li_2S, LiCl, and Li_3P.

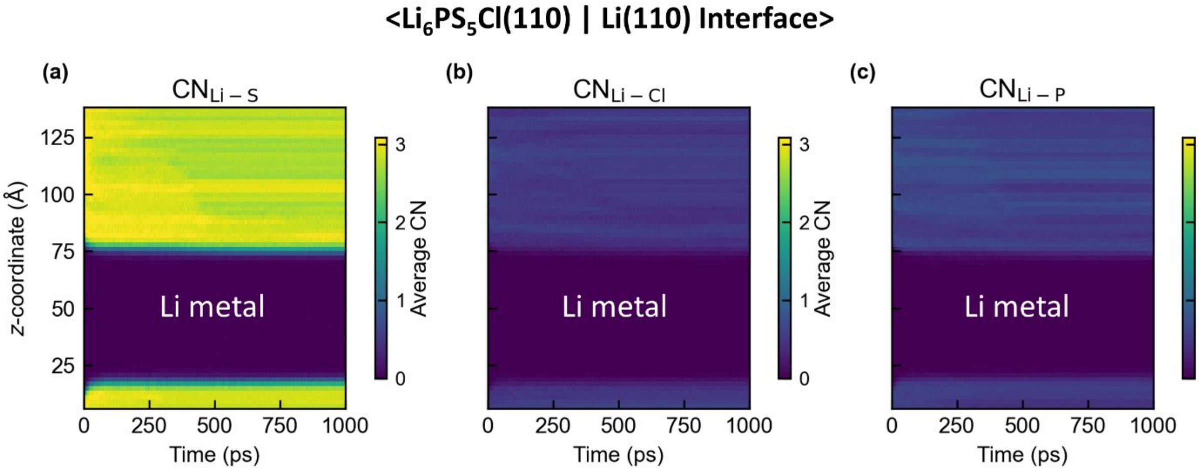

Figure S14. Time- and space-resolved coordination number analysis of the $Li_6PS_5Cl(110)$ | Li(110) interface. (a-f) Coordination number distributions averaged within each z-slice as a function of simulation time: (a) S around Li (Li-S), (b) Cl around Li (Li-Cl), and (c) P around Li (Li-P).

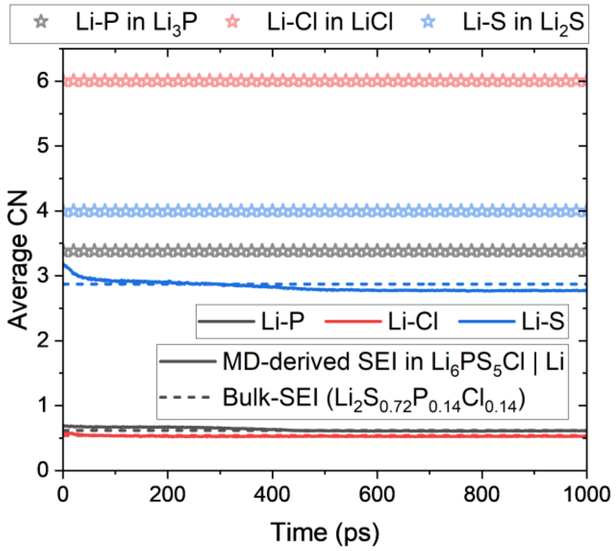

Figure S15. Time evolution of the averaged coordination numbers for Li-P, Li-Cl, and Li-S pairs calculated within the SEI region of the $Li_6PS_5Cl(110)$ | Li(110) interface, defined by 85 Å < z < 140 Å. The corresponding coordination numbers in the bulk-SEI phase ($Li_2S_{0.72}P_{0.14}Cl_{0.14}$) and in conventional decomposition products, Li_3P for Li-P, LiCl for Li-Cl, and Li_2S for Li-S, are shown for comparison.

I. Ionic conductivity and thermodynamic stability of $Li_6PS_{5-x}ClO_x$ as a function of anti-site defect ratio

Figure S16 summarizes the dependence of ionic conductivity and thermodynamic stability on the extent of S(4d)-Cl(4a) exchange (anti-site defect ratio, R_{as}) in undoped and O-doped $Li_6PS_{5-x}ClO_x$. Overall, O doping tends to lower the ionic conductivity across most of the R_{as} range, except at R_{as}=1.0.

The relative energy (ΔE) serves as a thermodynamic metric to estimate the probability of forming a specific R_{as} configuration under experimental conditions. For both undoped and O-doped compositions, the introduction of anti-site defects (R_{as} = 0.25, 0.5, and 0.75) increases ionic conductivity by approximately 2-3 orders of magnitude compared with the ordered structure (R_{as} = 0). However, the thermodynamic trends differ markedly between the two systems. In undoped Li_6PS_5Cl, structures with R_{as} = 0.25 - 0.75 are substantially more thermodynamically stable than the R_{as} = 0 case, implying a higher probability of occurrence. In contrast, O-doped $Li_6PS_{5-x}ClO_x$ shows only a weak dependence of thermodynamic stability on R_{as}, suggesting that multiple anti-site defect ratios likely coexist with comparable probabilities. As a consequence, the equilibrium distribution of anti-site defect ratios is expected to differ between the undoped and O-doped systems, necessitating a thermodynamically weighted evaluation of ionic conductivity to approximate experimentally relevant behavior.

To account for this thermodynamic stability, we calculated the effective ionic conductivity at 300 K by applying Boltzmann statistics. The weighted mean values and their standard deviations are presented in Figure 4(a) of the main text. The Boltzmann weight (w_i) for each anti-site defect ratio (i) was derived from its relative energy as follows:

$$w_i = \frac{\exp(-\frac{\Delta E_i}{kT})}{\sum_j \exp(-\frac{\Delta E_j}{kT})} ,$$

where i denotes the specific anti-site defect ratio R_{as}, k is the Boltzmann constant, and T = 300 K. Using these weights, the effective ionic conductivities (σ) was calculated as:

$$\sigma = \sum_i w_i \sigma_i .$$

Similarly, the weighted mean and standard deviation of the energy above hull (E_{hull}) were calculated for each phase (Li_6PS_5Cl, $Li_6PS_{4.75}ClO_{0.25}$, and $Li_6PS_{4.5}ClO_{0.5}$) using the same weights as those applied in the effective ionic conductivity calculation:

$$E_{hull} = \sum_i w_i E_{hull,i} .$$

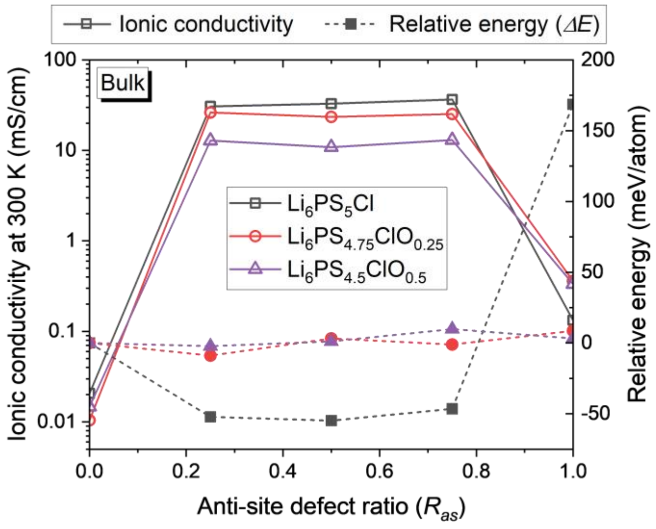

Figure S16. Ionic conductivity at 300 K and relative energy (*ΔE*) as a function of anti-site defect ratio in $Li_6PS_{5-x}ClO_x$ (x = 0, 0.25, and 0.5). *ΔE* values were obtained from 0 K energy-minimized structures.

J. Microscopic origin of enhanced Li-ion conductivity in O-doped bulk-SEI phases

To elucidate the microscopic origin of the enhanced ionic conductivity observed in the O-doped SEI, we performed complementary analyses, including radial distribution functions (RDFs), Li probability density mapping, and site-to-site Li hopping analysis, for the $Li_2S_{0.72}P_{0.14}Cl_{0.14}$ and $Li_2S_{0.68}P_{0.14}Cl_{0.14}O_{0.04}$ systems. Figure S17 presents the Li-anion RDFs for Li_2S, $Li_2S_{0.72}P_{0.14}Cl_{0.14}$, and $Li_2S_{0.68}P_{0.14}Cl_{0.14}O_{0.04}$.

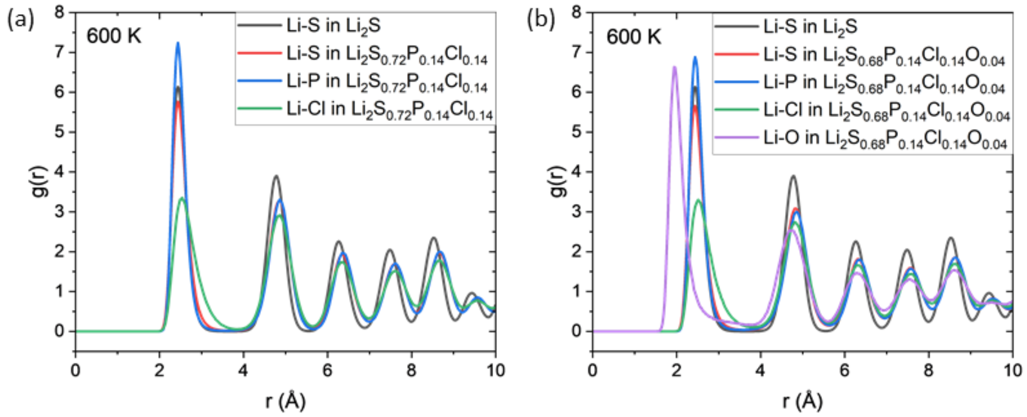

Figure S17. Li-anion radial distribution functions (RDFs) for (a) Li_2S and $Li_2S_{0.72}P_{0.14}Cl_{0.14}$, including Li-S, Li-P, and Li-Cl pairs, and (b) Li_2S and $Li_2S_{0.68}P_{0.14}Cl_{0.14}O_{0.04}$, including Li-S, Li-P, Li-Cl, and Li-O pairs.

To directly visualize how P/Cl/O-induced structural changes affect Li migration, we constructed three-dimensional Li probability density maps from the MD trajectories by accumulating Li positions on a regular grid, normalizing by the total number of Li samples, and smoothing with a Gaussian filter under periodic boundary conditions. As shown in Figure S18, $Li_2S_{0.68}P_{0.14}Cl_{0.14}O_{0.04}$ (panels d-f) displays noticeably more diffuse and interconnected Li density relative to $Li_2S_{0.72}P_{0.14}Cl_{0.14}$ (panels a-c) when viewed along all three orthogonal directions. In particular, regions of high Li probability density are preferentially observed near O atoms in the O-doped system, suggesting that oxygen-containing local environments facilitate Li transport pathways.

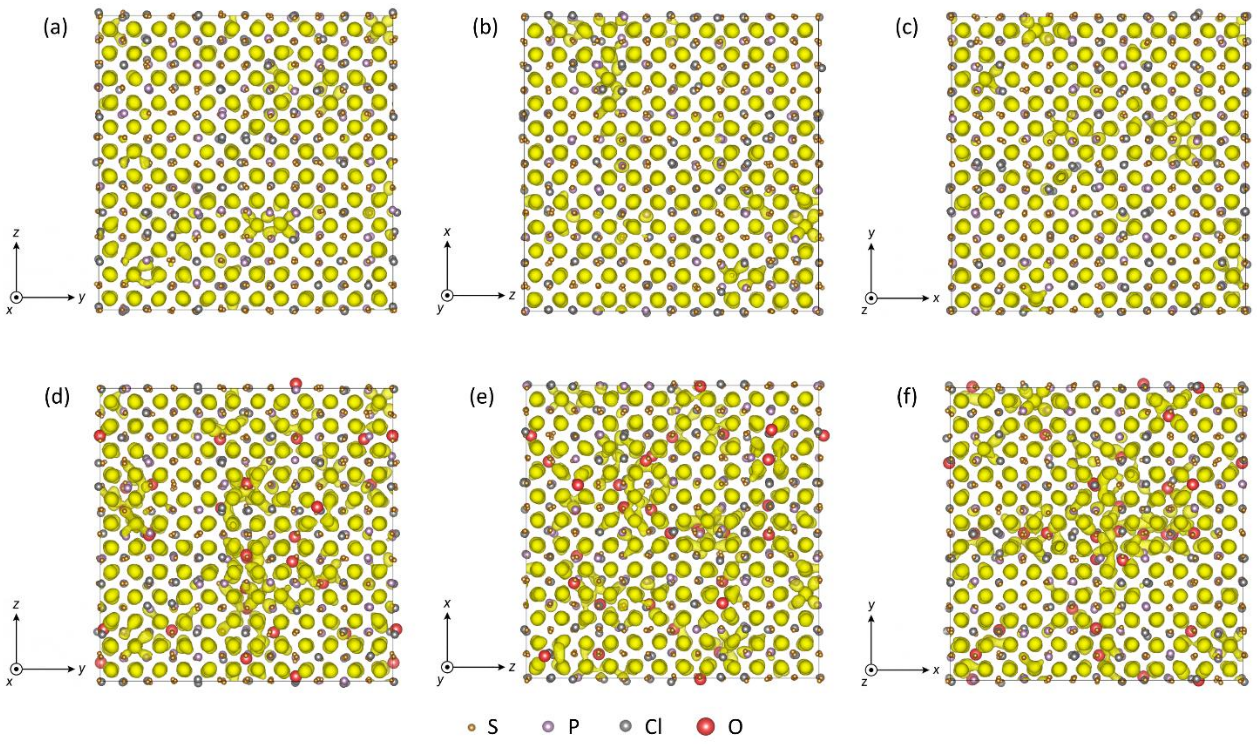

Figure S18. Li probability density maps of (a-c) $Li_2S_{0.72}P_{0.14}Cl_{0.14}$ and (d-f) $Li_2S_{0.68}P_{0.14}Cl_{0.14}O_{0.04}$ at 600 K. Panels (a-c) and (d-f) show the Li trajectories viewed along three orthogonal directions, perpendicular to the x-, y-, and z-axes, respectively.

To quantify the dynamical consequences of these structural changes, we performed a site-resolved Li hopping analysis based on the local maxima of the smoothed Li probability density. Reference Li sites were identified as density maxima, and in each MD frame, Li ions were assigned to their nearest reference site within a cutoff distance. A hopping event was recorded when an individual Li ion changed its assigned reference site, and the residence time was defined as the duration of continuous occupancy at a single site. As shown in Figure S19(a), the O-doped $Li_2S_{0.68}P_{0.14}Cl_{0.14}O_{0.04}$ system exhibits a significantly larger fraction of short residence times compared to $Li_2S_{0.72}P_{0.14}Cl_{0.14}$, indicating that O doping promotes more frequent and rapid Li site exchange. Consistently, the overall Li hopping frequency is higher in the O-doped system (Figure S19(b)). To clarify the local origin of this enhancement, Li sites were further classified according to the framework species located within 3 Å of each reference Li site. While P-containing environments show a moderate increase in hopping frequency in both systems, Li sites coordinated by O atoms exhibit a markedly enhanced hopping frequency that far exceeds that of O-free S-, P-, and Cl-containing environments. This site-resolved result clearly shows that oxygen-containing local environments strongly facilitate Li-site exchange. Taken together, the RDF, Li probability density, and hopping analyses provide direct evidence that O doping enhances Li ion transport in the SEI.

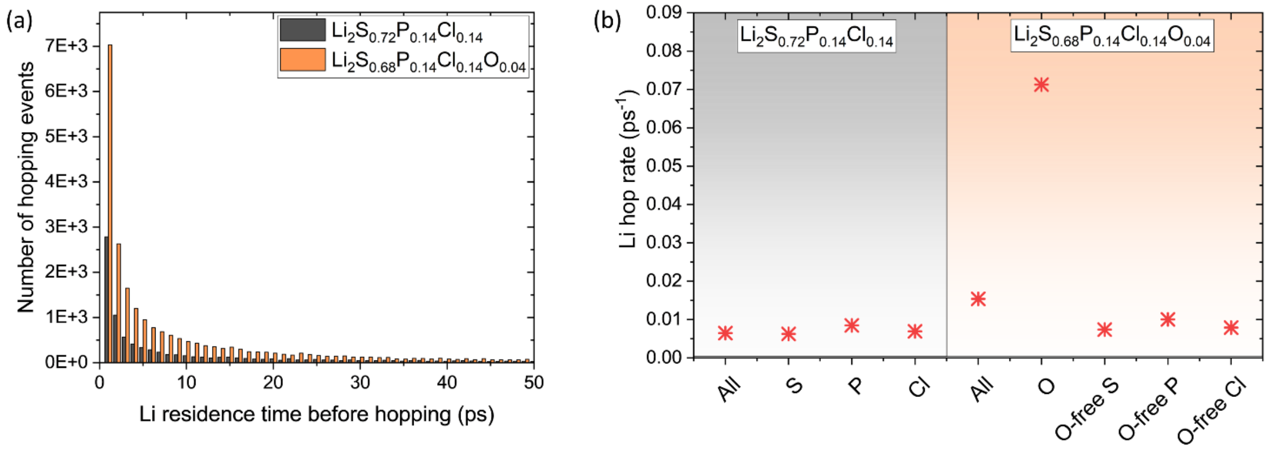

Figure S19. (a) Distribution of Li residence times before hopping for the $Li_2S_{0.72}P_{0.14}Cl_{0.14}$ and O-doped $Li_2S_{0.68}P_{0.14}Cl_{0.14}O_{0.04}$ systems. (b) Li hopping frequency for different Li-site environments. The hopping frequency was calculated as the number of observed hopping events divided by the total Li residence time. "All" represents all assigned Li sites in each system. S, P, Cl, and O denote Li sites with at least one corresponding framework atom within 3 Å of the reference Li site. For the O-doped system, O-free S, O-free P, O-free Cl denotes S-, P-, and Cl-containing Li sites without neighboring O atoms.

K. Arrhenius plots of Li-ion diffusion coefficient

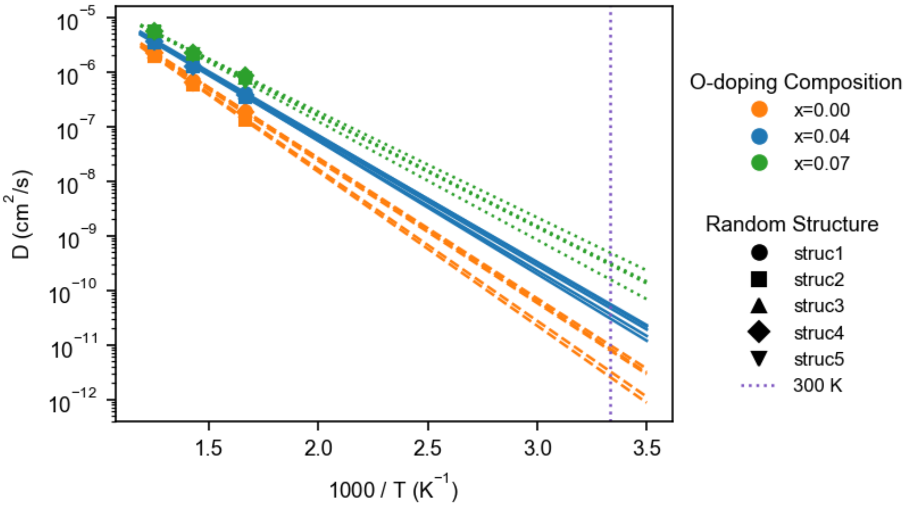

Figure S20. Arrhenius plots of Li-ion diffusion coefficients for $Li_2S_{0.72-x}P_{0.14}Cl_{0.14}O_x$ with x = 0.00, 0.04, and 0.07. For each composition, five randomly generated structures were simulated. Diffusion coefficients were calculated at 600, 700, and 800 K, and linear fits of log(D) versus 1000/T were used to extrapolate the diffusion coefficients to 300 K (vertical purple dashed line). Colors indicate composition, while symbols denote the individual randomly generated structures.

L. Electronic density of states of bulk-SEI phase

Electronic density of states (DOS) calculations were performed using VASP with the HSE06 hybrid functional. The plane-wave cutoff energy was set to 520 eV, and a Γ− centered 2×2×2 k-point mesh was used. The structures of $Li_2S_{0.75}P_{0.125}Cl_{0.125}$ and $Li_2S_{0.688}P_{0.125}Cl_{0.125}O_{0.062}$ are shown in Figure S21. These structures were constructed by substituting S atoms with P and Cl (and O) in a 2×1×2 supercell of Li_2S (48 atoms in total).

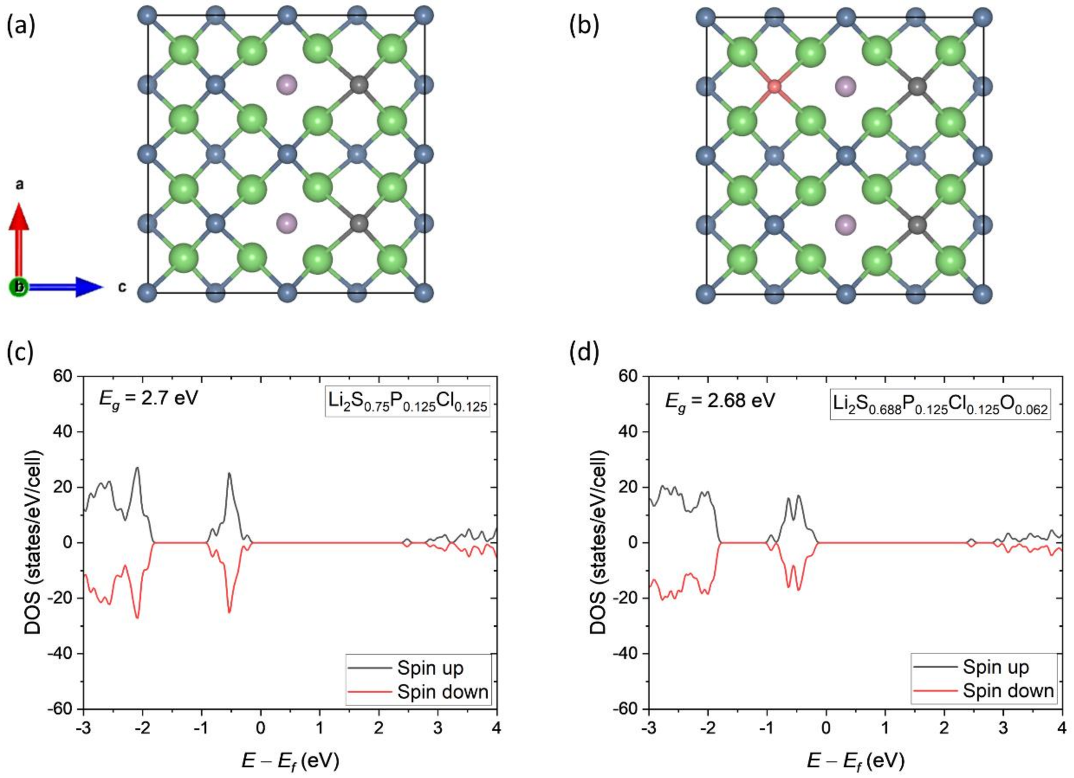

Figure S21. Atomic structures and electronic density of states of (a, c) $Li_2S_{0.75}P_{0.125}Cl_{0.125}$ and (b, d) $Li_2S_{0.688}P_{0.125}Cl_{0.125}O_{0.062}$, respectively. Li, S, P, Cl, and O atoms are shown in green, blue, purple, grey, and red, respectively.

To examine whether structural disorder can significantly alter the electronic passivation behavior, we additionally calculated the spin-polarized total DOS for two disordered structures extracted from high-temperature AIMD simulations, as shown in Figure S22. In the AIMD simulations, the system was heated from 300 to 2500 K over 5 ps and then maintained at 2500 K for 10 ps. Two representative snapshots were extracted after 9 and 10 ps of the 2500 K holding stage for subsequent DOS calculations. Compared with the relaxed crystalline $Li_2S_{0.75}P_{0.125}Cl_{0.125}$ structure, the AIMD-derived snapshots show only a slight reduction in the band gap and the DOS near the Fermi level remains negligible. This suggests that thermal structural disorder alone does not generate an extended electronic conduction pathway in the examined SEI model structures.

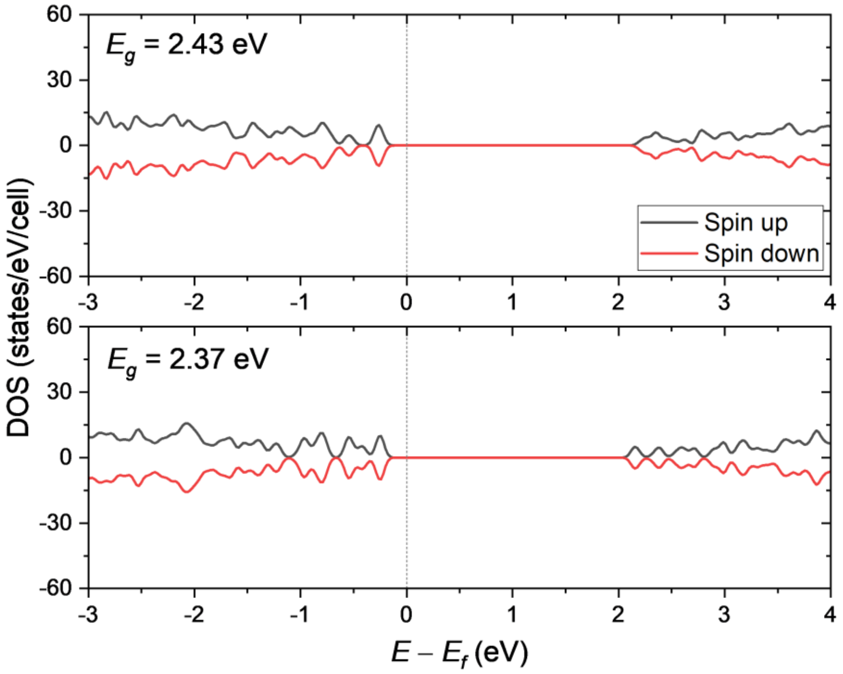

Figure S22. Spin-polarized density of states (DOS) for thermally disordered $Li_2S_{0.75}P_{0.125}Cl_{0.125}$ structures extracted after 9 ps (top) and 10 ps (bottom) of the 2500 K AIMD holding stage.

To further examine the possibility of defect-mediated electronic leakage beyond thermal structural disorder, we calculated the spin-polarized total density of states for Li-vacancy-containing configurations with the vacancy located near S, P, or Cl atoms (Figure S23). Upon Li-vacancy formation, additional defect-related features appear within the band gap in the spin-polarized DOS. However, the DOS at the Fermi level is negligible for all examined Li-vacancy configurations, indicating that Li vacancies do not generate metallic Fermi-level states.

Nevertheless, we note that the present calculations are based on representative defect configurations and do not exhaustively sample all possible amorphous local environments, defect concentrations, or extended defects such as grain boundaries. Such structural features may introduce additional localized states or modify hopping barriers between defect sites. Therefore, while the present DOS analyses support the absence of metallic Fermi-level states in the examined SEI models, further large-scale sampling and explicit hopping-barrier calculations would be valuable for a more complete assessment of defect-mediated electronic leakage.

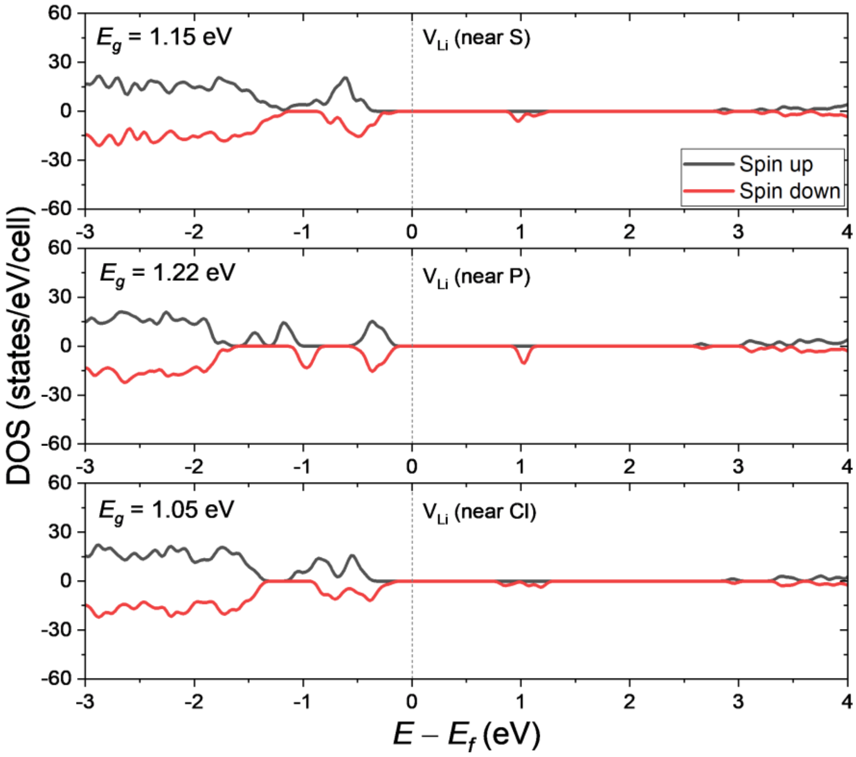

Figure S23. Spin-polarized density of states (DOS) for $Li_2S_{0.75}P_{0.125}Cl_{0.125}$ with a Li vacancy located near (top) S, (middle) P, and (bottom) Cl atoms.

M. Structural relaxation and phonon calculations

Structural relaxations and phonon calculations were performed using the SevenNet-based MLIP constructed in this work. The initial atomic structures of $Li_2S_{0.75}P_{0.125}Cl_{0.125}$ and $Li_2S_{0.625}P_{0.125}Cl_{0.125}O_{0.125}$ were first fully relaxed. Phonon calculations were subsequently conducted to assess the dynamical stability of the relaxed structures.

Phonon properties were calculated using the finite-displacement method as implemented in the *phonopy* package.[3,4] Atomic displacements with an amplitude of 0.005 Å were applied to generate the set of displaced supercells, and atomic forces for each displaced structure were calculated using the MLIP. Phonon band structures were then obtained using *phonopy,* as shown in Figure S24. The absence of imaginary phonon modes confirms the dynamical stability of the investigated SEI-related phases.

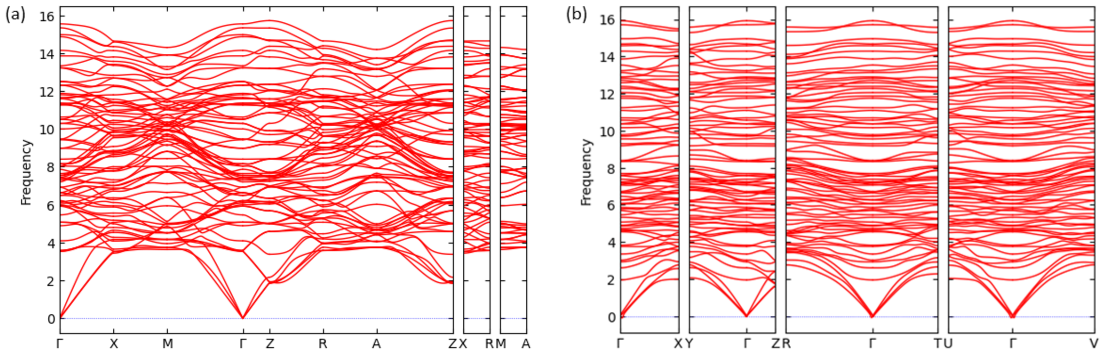

Figure S24. Phonon modes for (a) $Li_2S_{0.75}P_{0.125}Cl_{0.125}$ and (b) $Li_2S_{0.625}P_{0.125}Cl_{0.125}O_{0.125}$ using *phonopy* code and our constructed SevenNet-based MLIP.

N. Thickness-dependent resistance analysis of the SSE/SEI stack

Table S6. Ionic conductivities used for the SSE / SEI resistance analysis.

SSE / SEI	SSE ionic conductivity (mS/cm)	SEI ionic conductivity (mS/cm)
Li_6PS_5Cl (SSE) / $Li_2S_{0.72}P_{0.14}Cl_{0.14}$ (SEI)	31.76453	0.00164
$Li_6PS_{4.75}ClO_{0.25}$ (SSE) / $Li_2S_{0.68}P_{0.14}Cl_{0.14}O_{0.04}$ (SEI)	16.75574	0.01157
$Li_6PS_{4.5}ClO_{0.5}$ / $Li_2S_{0.65}P_{0.14}Cl_{0.14}O_{0.07}$ (SEI)	7.2888	0.08206

Table S7. Thickness-dependent R_{SEI}, R_{total}, and σ_{eff} calculated for the Li_6PS_5Cl (SSE) / $Li_2S_{0.72}P_{0.14}Cl_{0.14}$ (SEI) stack using L_{SSE} = 500 µm.

SEI thickness (nm)	R_{SEI} (Ω cm2)	R_{total} (Ω cm2)	σ_{eff} (mS/cm)
0	0.000	1.574	31.765
10	0.610	2.184	22.896
50	3.049	4.623	10.817
100	6.098	7.672	6.519
126	7.683	9.257	5.403
250	15.244	16.818	2.974
334	20.366	21.940	2.280
500	30.488	32.062	1.561
600	36.585	38.159	1.312

Table S8. Thickness-dependent R_{SEI}, R_{total}, and σ_{eff} calculated for the $Li_6PS_{4.75}ClO_{0.25}$ (SSE) / $Li_2S_{0.68}P_{0.14}Cl_{0.14}O_{0.04}$ (SEI) stack using L_{SSE} = 500 µm.

SEI thickness (nm)	R_{SEI} (Ω cm2)	R_{total} (Ω cm2)	σ_{eff} (mS/cm)
0	0.000	2.984	16.756
10	0.086	3.070	16.284
50	0.432	3.416	14.638
100	0.864	3.848	12.995
126	1.089	4.073	12.279
250	2.161	5.145	9.723
334	2.887	5.871	8.522
500	4.322	7.306	6.851
600	5.186	8.170	6.127

Table S9. Thickness-dependent R_{SEI}, R_{total}, and σ_{eff} calculated for the $Li_6PS_{4.5}ClO_{0.5}$ / $Li_2S_{0.65}P_{0.14}Cl_{0.14}O_{0.07}$ (SEI) stack using L_{SSE} = 500 µm.

SEI thickness (nm)	R_{SEI} (Ω cm2)	R_{total} (Ω cm2)	σ_{eff} (mS/cm)
0	0.000	6.860	7.289

10	0.012	6.872	7.276
50	0.061	6.921	7.225
100	0.122	6.982	7.163
126	0.154	7.013	7.131
250	0.305	7.164	6.982
334	0.407	7.267	6.885
500	0.609	7.469	6.701
600	0.731	7.591	6.595

References

1 H. Lee, H. Kim, S. Ji, K. Choi, H. Choi, W. Lim and B. Lee, *Advanced Energy Materials*, 2024, **14**, 2402396.

2 L. van der Maaten and G. Hinton, *Journal of Machine Learning Research*, 2008, **9**, 2579–2605.

3 A. Togo, L. Chaput, T. Tadano and I. Tanaka, *J. Phys.: Condens. Matter*, 2023, **35**, 353001.

4 A. Togo, *J. Phys. Soc. Jpn.*, 2023, **92**, 012001.
